# Cave morphology and human-mediated sediment deposition: Late Pleistocene to Holocene evolution of the cave floor at Panga ya Saidi, coastal Kenya

**DOI:** 10.1371/journal.pone.0347491

**Published:** 2026-05-20

**Authors:** Jennifer M. Miller, Ceri Shipton, Nikos Kourampas, Josep M. Parés, Hubert Vonhof, Mohammad Javad Shoaee, Arturo Cueva-Temprana, Makayla Harding, Alison Crowther, Dovydas Jurkenas, Doreen Mutoro, Jacopo Niccolò Cerassoni, Ricardo Fernandes, Christine Maroma, Emmanuel Ndiema, Nicole Boivin, Michael D. Petraglia

**Affiliations:** 1 Center for Social Sciences, Southern University of Science and Technology, Shenzhen, Guangdong, China; 2 Department of Archaeology, Max Planck Institute of Geoanthropology, Jena, Thuringia, Germany; 3 College of Asia and the Pacific, The Australian National University, Canberra, Australia; 4 Institute of Archaeology, University College London, London, United Kingdom; 5 Edinburgh School of Architecture & Landscape Architecture, University of Edinburgh, Edinburgh, United Kingdom; 6 Biological and Environmental Sciences, University of Stirling, Stirling, United Kingdom; 7 Geochronology & Geology, Spanish National Research Center for Human Evolution, Burgos, Spain; 8 Department of Climate Geochemistry, Max Planck Institute for Chemistry, Mainz, Germany; 9 Area de Prehistoria, Departamento de Historia, Geografía y Comunicación, Universidad de Burgos, Burgos, Spain; 10 School of Social Sciences, The University of Queensland, Brisbane, Australia; 11 Independent Researcher, Nairobi, Kenya; 12 Department of Biology, Loyola University Chicago, Chicago, Illinois, United States of America; 13 National Museums of Kenya, Nairobi, Kenya; 14 Institute of Energy and Environmental Technology, Jomo Kenyatta University of Agriculture and Technology, Nairobi, Kenya; 15 School of Science and Environment, Griffith University, Brisbane, Australia; 16 Australian Research Center for Human Evolution, Griffith University, Brisbane, Australia; 17 Human Origins Program, National Museum of Natural History, Smithsonian Institution, Washington, United States of America; Universita degli Studi di Milano, ITALY

## Abstract

Panga ya Saidi (PYS), a cave situated in a forested upland near the coast of Kenya, contains an archaeological record that spans at least 78,000 years and is crucial for understanding the evolution of *Homo sapiens* populations in eastern Africa. Here we report on new systematic excavation, three-dimensional scanning, radiocarbon dating, and geoarchaeological analyses that provide insights on sedimentary dynamics, occupation patterns, and site-formation processes at PYS. Our data show that while unroofed parts of the cave probably contain the deepest stratigraphies, these are likely to be dominated by colluvial influx and yield limited cultural deposits. Sheltered zones near the chamber edges exhibit better stratigraphic integrity and higher artifact densities, with deposits exceeding 5.5 meters in thickness in the main excavation area. In this area, marked shifts in occupation intensity and sediment sources enable us to reconstruct the evolution of the inhabited cave space. Our findings underscore PYS as a key site for understanding human behavior, adaptation, and environmental interactions from the Late Pleistocene to the Holocene, with implications for broader interpretations of the evolution of *Homo sapiens* populations in eastern Africa.

## Introduction

Eastern Africa is a key region for understanding the evolution of *Homo sapiens* behavior. A select number of archaeological sites, primarily situated inland across Kenya and Tanzania, including Enkapune ya Muto [1], Lukenya Hill [2], Nasera [3], Mumba [3], and Kisese II (see summary in [4]), have shaped our knowledge of Pleistocene–Holocene cultural transformations in this region. Original excavations at these sites occurred decades ago, before the availability of advanced methodologies such as microstratigraphy, digital data recording, and geochemical/ magnetostratigraphic analyses. Recent re-investigations at these locations (e.g., [5–9]) have employed modern techniques to enhance data collection and interpretation, exemplifying the importance of fine-grained archaeological research.

Panga ya Saidi (PYS), situated near the coast of Kenya, has emerged as a new and crucial site for understanding human evolution in Africa. With a consistent record of occupation over 78,000 years, PYS provides unparalleled insights into human occupation in a tropical upland setting during the Late Pleistocene–Holocene, across different socioecological regimes, from hunting and foraging to food production. Furthermore, the coastal location of PYS extends the better-known inland archaeological record, enhancing our understanding of behavioral adaptations in diverse environments. Previous research at PYS has described nineteen archaeological layers in the upper 3.5 meters (m) of sediment fill [10], with a series of more than 30 Bayesian-modelled radiocarbon and optically stimulated luminescence ages revealing successive episodes of human activity during each of the last five Marine Isotope Stages (MIS).

While initial publications have outlined the PYS archaeological sequence as a whole, detailing its chronological evolution and underscoring its unique features (e.g., [10–13]), descriptions of the cave sediments have offered a broad overview rather than detailed analysis, particularly for the markedly anthropogenic deposits of the upper levels. To address this limitation, we initiated new fieldwork with the aim of applying digital recording techniques to scrutinize processes of site formation at PYS, focusing on the upper section of deposits as well as the evolving cave morphology. Three-dimensional (3D) scanning of the cave walls, total station recording of artifacts, and new radiocarbon ages offer a fresh and detailed perspective on site formation and stratigraphic integrity at PYS. These findings serve as a reference point for future work at the site, and contribute to a broader understanding of human behavior during the terminal Pleistocene and Holocene in eastern Africa.

## Materials and methods

### Previous fieldwork at Panga ya Saidi

**Fig 1:**
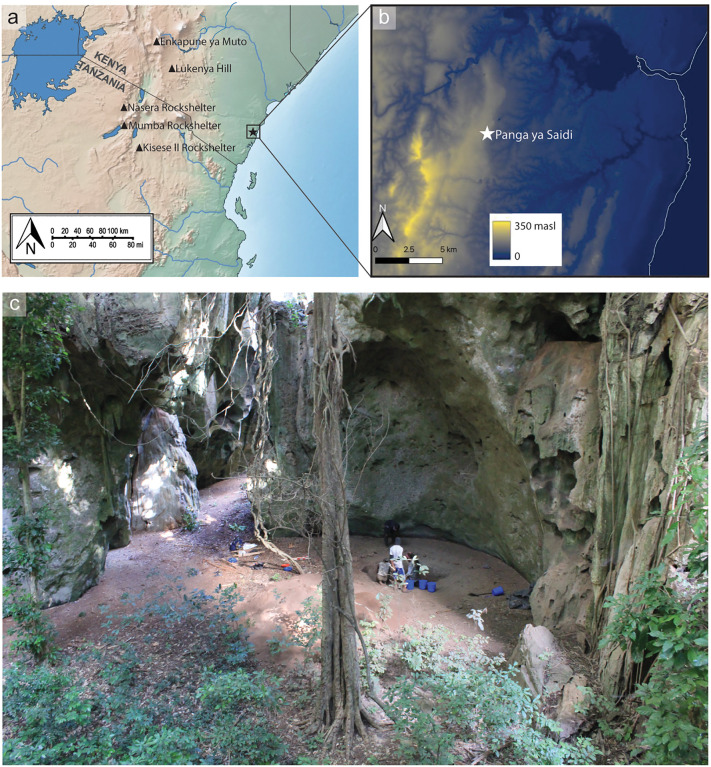
Panga ya Saidi site location and excavation areas. a) Regional map showing the location of PYS in relation to other sites mentioned in the text. Author created map using base map data from Natural Earth (public domain); **b)** Local elevation map, elevation data derived from the JAXA ALOS World 3D 30 m Digital Elevation Model (AW3D30), Version 4.1 [14], and shoreline data [15]. Map created by the authors complying with JAXA Terms for Use. **b)** Photograph of main excavation area, under shelter overhang.

Panga ya Saidi (3°40’41.4“S 39°44’09.4”E), near Kilifi, coastal Kenya, is situated in Mid-Jurassic limestone nestled in a narrow strip of Zanzibar–Inhambane coastal forest, approximately 15 kilometers (km) from the coast (Fig 1a). Perched on an east-facing escarpment at the fringes of the Dzitsoni Uplands, PYS commands a vantage point overlooking the coastal plains (Fig 1b). Although most of the cave is unroofed due to ceiling collapse and erosion, some sections, including the main excavation area (Fig 1c), retain an overhang sufficient to form a dripline.

Although initially recognized as a cave with archaeological potential in 1975 by Robert Soper [16], PYS was not excavated until 2010, as part of the Sealinks project [17,18], which aimed to trace early trade networks across the Indian Ocean [19,20]. Excavation was initiated at two locations within the cave, with a 2 x 1-m trench at each. Trench 1 was located under a dripline near the entrance; Trench 2 was approximately 100 m to the west of Trench 1, deeper into the cave, under an arch [17]. Both locations yielded finds; however, Trench 2 had a sudden and dramatic decrease in density of lithics and pottery below the surface layer, while Trench 1 maintained a high abundance of material culture throughout. This prompted continued excavation in this high-yield, near-entrance area over three seasons (2011, 2013, 2017). During that time, the main excavation area was divided into seven contiguous trenches (# 1, 3–8) of variable shape, dimensions, and depth [13].

Controlled excavations over these three field seasons reached a maximum depth of 3.5 m below the cave floor without reaching bedrock [10,21]. At the conclusion of the 2017 season, excavators employed a battery-powered hand drill to auger into the trench floor, achieving an additional depth of 0.85 m (total depth over 4.3 m) with no obstruction that might indicate bedrock. The depth of the cave fill in this main excavation area, and therefore the potential for an even earlier archaeological record within the cave, remains unknown.

### Geological context

We initiated new fieldwork at PYS in 2020 to better understand the cave’s morphology (especially the part of the cave concealed by deposits), site formation and depositional processes, as well as to identify promising areas within the cave for future excavation. We deployed geological and geomorphological survey, digital mapping of the cave and wall morphology, and augering of the cave fill.

The cave was remapped using a Leica © Blk360 robotic total station that captures panoramic imagery and records 360,000 points per second to create a point cloud, with accuracy within 6 millimeters (mm) at a distance of 10 m, or 8 mm at 20 m. To increase the resolution around the main excavation area, we cleared light vegetation and increased the number of scans. Individual scans carried out within separate cave chambers were combined and processed in Autodesk © ReCap Pro. This scale model formed the basis for new, more accurate cave maps. Further, the processed point cloud was exported as an.e57 file and imported into Cloud Compare © where the cave system point cloud was meshed and simplified, and then exported as a wavefront.obj mesh file. The.obj mesh was imported onto QGIS © 3.28.15 ‘Firenze’ and Meshlab © 2023.12 and processed to extract cross sections of the cave walls, as well as roof and wall morphology, even in dark chambers.

### Anthropogenic inputs

To increase our understanding of human activity during the deposition on the upper part of the PYS stratigraphy, a new 4 x 1 m excavation trench was opened along the northern edge of the previous excavation area. In order to systematize the recovery of finds, we divided the area into rectilinear vertical units, with each unit assigned a unique alphanumeric code. The main excavation in 2020 thus comprised three northern units (J13, K13, L13). Excavation proceeded with the single-context method within grid squares, subdivided into 10-centimeter (cm) spits for thicker contexts. Context boundaries were identified based on detectible differences in sediment color, texture, compaction, inclusions, and archaeological finds. Individual artifacts were recorded in situ with a Leica Viva TS16 robotic total station. Ceramic, lithic, and large faunal specimens (≥2 cm), as well as any aquatic shells, beads, ocher, teeth, and other special finds regardless of size, were plotted. Unmodified landsnail shell was sampled but not plotted as it was abundant, and likely to be partly the result of natural accumulation (e.g., aestivation; although consumption of Achatinids is well documented by Late Pleistocene/Holocene foragers in Africa [22], including in Kuumbi cave on nearby Unguja island (Zanzibar) [23]). All sediment was dry-screened on-site through a 3-mm mesh.

Vertical spatial analysis of the piece-plotted finds was conducted to identify concentration hotspots by depth. Using the Kernel Density method [24] in ArcMap 10.5.1 @ArcGIS, we created individual density models for lithics, bones, ceramics, and shells. We chose the Kernel bandwidth of 0.192 m based on the spatial variant of Silverman’s Rule of Thumb that offers the best density estimates for a given area [24]. To calculate the individual categories, we used a fixed bandwidth of 0.18 m, which allows the comparative assessment and differentiation of category distributions. The outcomes were interpolated by applying bilinear interpolation.

Chronostratigraphic control was based on ^14^C dating of artifacts (such as ostrich eggshell beads) or associated material (wood charcoal, shell, charred seeds) from the same context. We further employed the software package OxCal v. 4.4.4 [25] for Bayesian chronological modeling (OxCal model available in S1 File). Radiocarbon calibration relied on IntCal20, SHCal20, and Marine20 calibration curves [26–28]. Since PYS is located within the range of the Intertropical Convergence Zone, which may have varied in the past, we calibrated terrestrial samples using a mixed IntCal20 and ShCal20 curve with unknown mixing proportions (by setting a uniform prior in OxCal between 0 and 100). Marine shells may exhibit a marine radiocarbon reservoir effect [29]. As we lack local and temporally proximate estimates of marine ΔR (local deviation from global Marine20), this was estimated following the method described in Fernandes et al. [30]. A global Bayesian smoothed surface was generated for ΔR using data from the Marine Reservoir Database. From this we estimated the local ΔR as −45 ± 44 ^14^C yrs.

A Bayesian model of absolute age estimates was constructed from Layers 1–8, as well as for a human burial at the top of the sequence from which DNA was recovered [31]. This complements the revised Bayesian model for the deeper Layers 9–19 [21]). Samples from each stratigraphic unit were grouped as phases separated by boundaries following OxCal terminology. In the model, we set sharp (Boundary) or more gradual (Sigma_Boundary) transitions between layers and features on the basis of stratigraphic evidence. For sharp transitions with observable stratigraphic hiatuses we set adjacent boundaries. Our radiocarbon measurements may introduce some chronological biases due to stratigraphic intrusions, limited pretreatment (one charred sample was subject to acid-only pretreatment instead of the standard acid-base-acid pretreatment), old wood effect, carbonate contamination, and deviations from the estimated marine ΔR [32–36]. Although we expect that our radiocarbon measurements are accurate and that the set chronological sequence is reliable overall, to mitigate the influence of potential dating inaccuracies, we employed an outlier general model [37] by assuming that temporal outliers followed a Student’s t distribution with 5 degrees of freedom, and employed a wide temporal scale (0–10,000 years). To each sample, we assigned a prior probability of 5% that it was an outlier.

Human occupation intensity was calculated using an adapted form of the lithics per spatial unit per year index [38]. Mid-points of start- and end-age estimates of Layers 1–8 were taken from the Bayesian model to calculate layer duration. The number of lithic artefacts and liters of sediment excavated (counted as filled 10 liter buckets during excavation) from Trenches 1–7 were totaled for each layer. The advantage of this index is that lithics provide an independent measure of human presence, circumventing the issue of an age relationship with radiocarbon sample failure that biases ^14^C dates against older population estimates [39].

No samples for micromorphological analysis were collected from the 2020 excavation due to COVID restrictions prematurely halting the excavation season. Four thin sections from the Trench 4 2013 sample set (n = 10), correlative with the 2020 stratigraphy, were used as a micromorphological reference. The methodology of this earlier investigation is described in [10,12]. In brief, in view of the large dimensions and stratigraphic complexity of the 2013 Trench 4, sampling was at a reconnaissance scale, concentrating on layer boundaries and distinctive features. Undisturbed sediment samples were collected from the profile in clear polyurethane boxes. Sample boxes were labelled, photographed, and plotted on the profile drawing before removal from the profile. Ten of these samples were processed for sectioning and analysis at the Thin Section Micromorphology Laboratory, University of Stirling and at the Institute of Geography, University of Edinburgh (sample code PYS). After air-drying and impregnation with polyester (polylite) resin following standard procedures (http://www.thin.stir.ac.uk/), ca. 30 μm-thick, uncovered, large-format thin sections (7.5 × 11 cm) were manufactured from the hardened blocks. Thin sections were observed with a polarising microscope at magnifications of ×12.5 to ×400, using plain polarised (PPL), cross-polarised (XPL) and oblique incident light (OIL), and described following the terminology of Stoops [40] and Stoops et al. [41]. The relative abundance of sediment/soil components was estimated using standard semi-quantitative estimation charts. Key sediment constituents larger than ca. 50 μm (principal mineral grains; pedoclasts (soil fragments); biogenic particles, etc.) were point-counted using a 0.5 x 0.5 cm grid printed on a clear acetate overlay. As discussed below, lateral facies variation and the location of the 2020 excavation across the overhang-dripline, a dynamic hydrological, sedimentological, and ecological boundary that likely retreated during the timeframe of sediment deposition, qualify the applicability of this earlier microstratigraphic reference to the 2020 stratigraphy. Being closer to the cave wall, the 2013 stratigraphy represents a more ‘inner-rockshelter’ setting than the 2020 stratigraphy.

## Results

### Cave geology, morphology, and sediment fill

Panga ya Saidi formed in Kambe Limestone, an over-300-m-thick shallow-marine limestone formation of Mid-Jurassic age (Late Bajonian–Bathonian) [42,43]. This limestone formation stretches for several kilometers along the shore, between the hilltops on earlier (Triassic) sandstone (e.g., Cha Simba, Kiwara) and the lower, Quaternary surfaces of the coastal plain, mainly formed on later Mesozoic siliciclastics and Quaternary marginal marine and alluvial sediments [42,44]. In the Dzitsoni Uplands, Kambe Limestone generally dips gently (≤20°) to the ESE (seawards), and is bounded to the east by a low, locally steep, NNE–SSW trending, shore-parallel escarpment, interpreted as an eroded and backstepped fault-line scarp.

**Fig 2:**
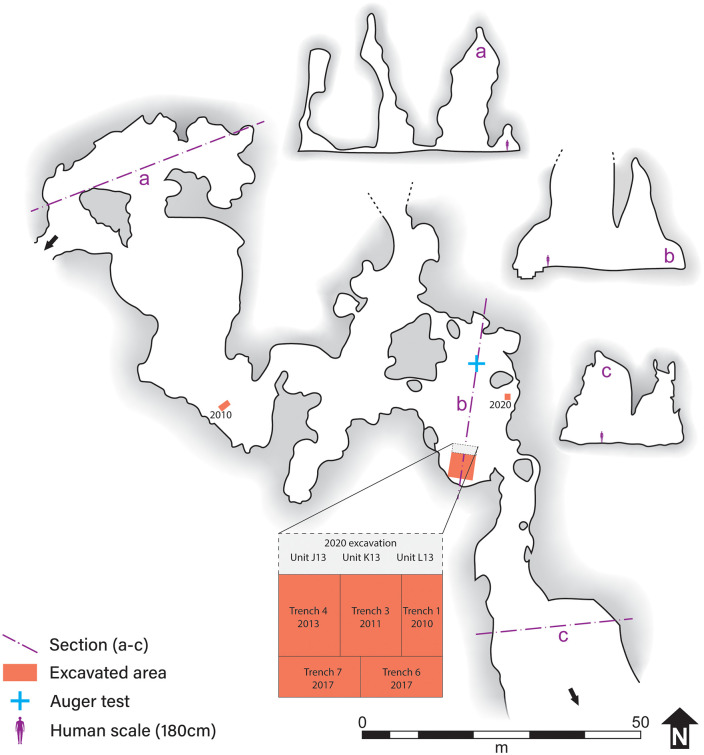
Plan of the southern part of the PYS cave system, including the excavated areas (red rectangles), and auger test. a–c) Cross section of selected locations, with human scale (180 cm) for reference. Cave entrances (to the south and northwest) are marked with arrows. The cave system continues north of the map edge.

The site itself is situated on the footwall of this inferred fault. It comprises interconnected chambers covering over ca. 12,000 m^2^, and aligned predominantly N–S, transversely to the escarpment. Many smaller chambers and alcoves within the cave complex follow NNE–SSW and ENE–WSW directions, coincident with the regional tectonic fabric and faults and joints in the limestone in and around the cave (Fig 2).

**Fig 3:**
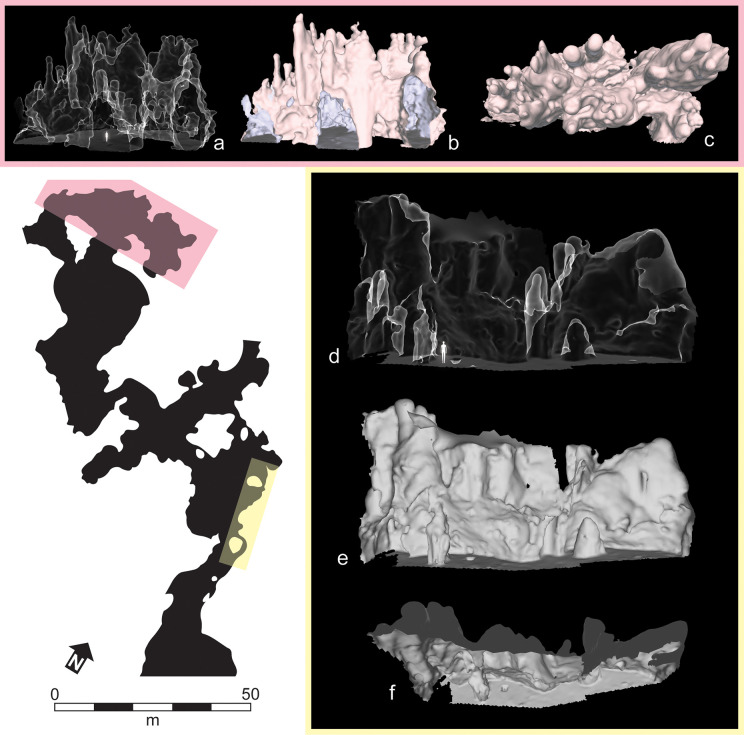
3D models of selected wall sections within PYS. Northern (pink, a–c) and central (yellow, d–f) eastern walls. Each section was represented using x-ray style (a, d) and radiance scaling rendered shaders (b–c, e–f). The northern section shows a cave chamber with three separate openings and a roof consisting of a series of cupolas. The central east section displays similar cupolas with sporadic stalagmites and stalactites.

Today, PYS is accessed from the escarpment and several steeper openings to the surface. The traversable part of the cave consists of large, interconnected, chambers with steep, vertical to subvertical walls. These chambers are unroofed to variable degrees, which has generated an environment akin to that of a karstic canyon, open to sediment and organisms from the overcave surface (Figs 2 and 3).

### Cave morphology and speleogenesis

The accessible part of PYS comprises three distinct altitudinal levels, overall spanning an estimated 10–12 m. Although the 3D morphology of the cave is partly obscured by sediment fill and ceiling collapse, the presence of perched interchamber openings (fenestrae) and remnants of horizontal phreatic tubes 4–8 m above the present cave floor suggest that PYS is a multilevel cave that evolved through several phases of speleogenesis.

**Fig 4:**
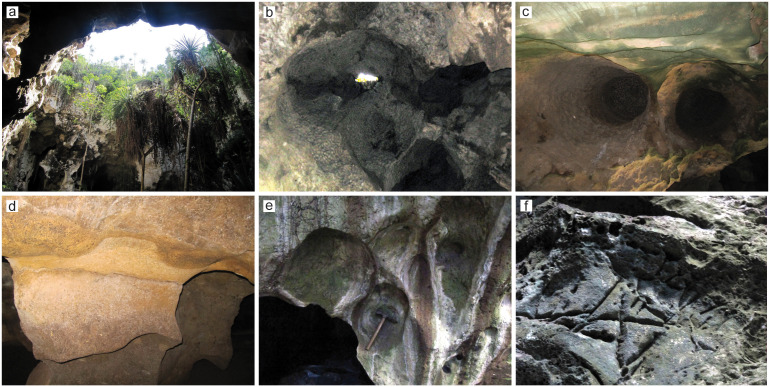
Chamber and wall morphology in PYS. a) Unroofing resulting from ceiling collapse. b) A cluster of deep, tapering-up cupolas with densely scalloped surfaces in one of the rear alcoves.The cupola at the center of the photograph has been breached and is open to the overcave surface. **c)** Meter-scale cupolas. **d)** Meter-scale cusps on alcove wall. **e)** Hemispherical hollows on bedrock limestone, perhaps resulting from condensation corrosion. **f)** Centimeter-scale grooves ca. 3.5 m above the northwestern entrance to the cave.

Ceiling collapse has unroofed much of the cave. In those sections that retain a ceiling, such as smaller side-chambers radiating off the main ones, the prevailing cavity morphology is elliptical to circular in plan and ovoid in cross section, with the exposed cavity height apparently equaling or exceeding their diameter. The high, apsed ceilings are formed by single or coalescent cupolas (vertical cavities of tubular shape that taper upward: Figs 3 and 4). Where the roof is retained, cupolas and other domed cavities are preferred sites for bat and owl roosting. Condensation corrosion, driven by the roosting bats, may have further modified and deepened cupolas and other ceiling cavities (c.f. [43]). Cupolas that open out to the surface are often used by hyraxes (Procaviidae). Bat guano continues to accumulate on the floor of many roofed chambers, while hyrax excrement accumulations occur in less accessible but unroofed parts of the cave.

Once considered to form as epiphreatic cavities above a temporarily elevated groundwater table (e.g., [43]), cupolas are now thought to originate from ascending flow in confined aquifer conditions [44]. Alongside the predominantly vertical development and tapering-up morphology of chambers and cupolas, parts of the cave preserve numerous speleomorphs (e.g., anastomosing half-tubes, vertical feeders, spongiform pores) collectively matching the ‘hypogene suite’ [45,46]. Overall, chamber and wall morphology indicates that PYS originally formed as a hypogene cave, by rising flow in a deep, confined aquifer. Large idiomorphic calcite crystals in karstic cavities and solution-enlarged fractures are also consistent with a hypogene (low-temperature hydrothermal) phase of cave evolution. Geological evidence from the cave itself, and the manifest unrelatedness of PYS with the Late Quaternary–present hydrogeological conditions, therefore, strongly suggest that PYS originated as a hypogene cave before regional uplift (perhaps due to the updoming of the distal flanks of the East African Rift System since the Late Tertiary) exhumed it and exposed it to erosion. The undermining of the cave ceiling in earlier hypogenic conditions may have conditioned ceiling collapse during the Quaternary exhumation of PYS (as also observed in much younger coastal caves in the region: [47]). Unroofing through collapse is ongoing, as demonstrated by the presence of large roof-fall blocks on the cave floor. Roof fall is aided by tree root bioerosion of the limestone and the episodic dislodgment of limestone blocks by trees felled during storms.

In PYS, stalactites and flowstones are generally encountered in their original growth position and linked to water conduits in the ceiling and overhangs. Some stalagmites are also positioned in places that retain a roof or overhang, with a few stalagmites actively growing. However, large exposed and weathered stalagmites in unroofed parts of the cave, and broken speleothems, suggest that at least some of the speleothem growth predates unroofing. Most speleothems are degraded, with calcite dissolution, neomorphism into phosphates and unknown powdery minerals (possibly gypsum), and colonisation and bioerosion by blue-green algae and lichens being common macroscopic alterations.

The regional depth of speleogenesis in Kambe Limestone (over 40–50 m), predominantly vertical development of (the exposed part of) chambers, and the presence of an > 20 m deep sinkhole a few meters east of the southern cave entrance suggest that, in parts of the cave, the sediment fill may extend meters beneath the present cave floor. This has important implications for the archaeological and palaeoenvironmental potential of PYS.

### Deposits

Deposits in the unroofed parts of PYS are markedly heterogeneous. Sediment facies on the cave floor vary across meters, from wall-adjacent parts to the center of unroofed chambers, to centimeters, especially around hydrological and ecological boundaries (e.g., driplines and under active stalactites, where dripping water washes away fine particles and concentrates pebbles in drip-point lags), owl nests, with accumulations of droppings and vertebrate bones, hearths, and other focal points of human activity. Bat guano accumulates in roofed side chambers, some of which are ancestral burial sites, marked by palisade structures at their entrance—where access is sporadic and regulated by ritual.

The central parts of large chambers, where the cave opens to the sky, are densely vegetated. In these parts of the cave, the topsoil is loose, root-bioturbated sandy silt with a spongy texture, usually under a thick layer of leaf litter. This spongy topsoil extends across the majority of the large chambers, especially in the northern part of the mapped cave (Fig 2). Facies variation at a similar scale was observed in the upper (Upper Pleistocene–Holocene) part of the stratigraphy, with higher-order centimeter to meter-scale facies present within the identified layers of the 2013 excavation (Trench 4) [10,12].

The auger test was located at the unroofed part of the southern chamber, approximately 5 m from the eastern wall (Fig 2). The augered deposits were consistently dark reddish-brown clayey silt, devoid of artifacts, bones, and shells. The first few centimeters were loosely packed and porous, but the combination of high clay content and moisture made it impossible to reach past a depth of 1.5 m. Beyond a gradual increase in clay content with depth, no other compositional or textural differentiation was identified. A shallow test trench a few meters from the auger test contrasted sharply with the auger record. Like the deposits in the main excavation area, the deposits of this test trench were drier, siltier, and more firmly compacted than the auger ones, and contained abundant artifacts and ecofacts.

### Stratigraphy and human habitation record

**Fig 5:**
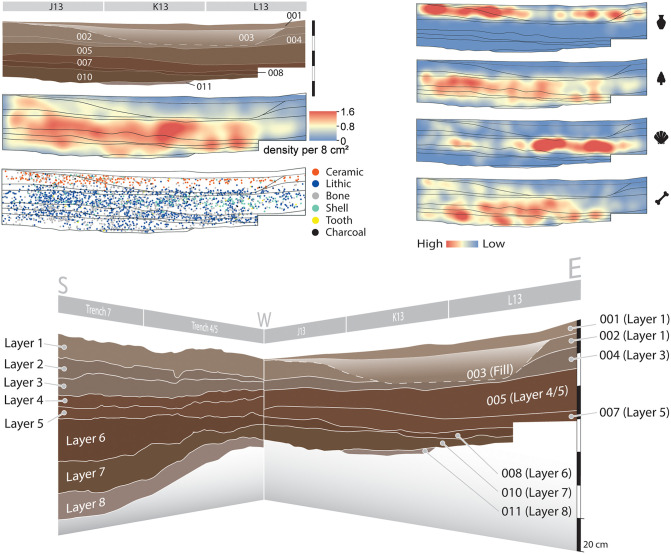
Stratigraphic profile of the main excavation area in 2020. Top left: North-facing view of newly excavated area (2020), find density per 8 cm² estimated using the Kernel Density method in ArcMap 10.5.1, and plotted finds by material, recorded *in situ* with a Leica Viva TS16 robotic total station, (visualized in ArcGIS). Top right: relative abundance (find density per 8 cm^2^) of ceramics, lithics, shell, and bones, visualized using the Kernel Density method in ArcMap 10.5. Bottom: Western and northern wall sections, and correlation with previously identified layers. For detailed description of contexts refer to Table 1. All scale bar segments are 20 cm.

The main excavation units in 2020 formed a block measuring 1 x 4 m, with excavations reaching a maximum depth of 77 cm below surface. We recorded the 3D location of more than 3,000 finds within this space, and correlated the newly excavated contexts with the previously established layers (Table 1, Fig 5).

**Table 1:** Contexts from the 2020 excavation. Description and correlation with previously identified layers, in stratigraphic order.

Context	Description	Layer
**001**	The upper few centimeters of sediment were brushed away as overburden. Very loose (soft packed), dark yellowish brown (10YR 3/4) lightly-sandy silt with occasional carbonate nodules. Contained leaf litter and scattered backfill. Random small finds including lithics, ceramics, bone, and shell.	Layer 1
**003**	Revealed directly under 001 and onlapping 002, this context is a lighter-colored, soft packed ashy sediment that represents a combustion area. Loose, light bluish grey (GLEY2 5B 7/1) lightly-sandy silt with moderate moisture content and frequent small charcoal inclusions, but overall homogenous. The top of Context 003 is slightly oval, but irregular with no clearly defined boundaries or cut, and fading very gradually into 002 and 004. Based on the slight tapering towards the bottom of 003, it likely postdates 002. Ceramic finds are present within 003: they are more abundant towards the edges and bottom, and generally less frequent than in 002.	Fill
**002**	Slightly more compact than 001, and visibly darker in color (although of the same Munsell value). Soft, dark yellowish brown (10YR 3/4) sandy silt with moderate moisture, occasional carbonate nodules and minor root disturbance. Located towards the northern and western edges of the main excavation block. Abundant microfauna and pottery.	Layer 1
	No context identified that corresponds with Layer 2.	Layer 2
**004**	A thin deposit that begins immediately below 002 and 003. Soft, dark yellowish brown (still 10YR 3/4) silt with moderate moisture, and increased presence of roots. Sediment composition is similar to 002, but with a noticeable reduction in the frequency of pottery and land snails, and few to no micromammal remains.	Layer 3
**005**	Separated from Context 004 by the presence of 4 large pieces of roof fall on a horizontal plane, and lithics lying flat, but otherwise the sediment matrix is similar. Firm, moderately dry, reddish brown to light reddish brown (slightly mottled from 5YR 4/3–5YR 5/3 and 5YR 6/3) sandy silt with minor rooting. There are minor shifts in color throughout 005, from 5YR 4/3–5/3–6/3, but no clear boundaries to indicate different horizons. Rare ceramics (n = 3), which may be intrusive. Rare micromammal remains but noticeable increase in large mammal bones and teeth. Abundant land snails and also marine shells. Increased lithic density, with many large lithics all lying on a horizontal plane. This Context is thicker than 10 cm and so was divided into 10-cm subsections (005A, 005B, 005C, 005D).	Layer 4/5
**006**	A localized context in the north-eastern part of the trench (not visible in the presented stratigraphic profile). Firm dark reddish brown (5 YR 3/4), damp sandy silt. Its borders are gradual and diffuse, but it appears roughly circular and extends further to the north and east (out of the excavated area). Finds include lithics, ceramic, shell, and bone. Overlain by 002, and possibly contemporary with 004.	
**007**	Underneath 005, from which it is separated by an indistinct boundary. A homogenous, moderately compacted, dark brown (7.5 YR 3/3) silt. Lower density of finds as compared to 005. Its upper part is similar to 005, with abundant chert and limestone flakes, and some marine shells. The lower part of 007 contains (mostly small) quartz lithics and burnt bones.	Layer 5
**008**	007 grades gradually into 008, a thin deposit of dark brown (7.5 YR 3/2, 3/3) silt with moderate moisture content. The change in context is based on the difference in find density (fewer snails and no marine shells); 008 may be broadly contemporaneous with 007. The base of 008 is marked by the appearance of gravels.	Layer 6
009	This context number was assigned to units on the southern side of the main excavation area, and is not present in the illustrated profile. Context 008 directly contacts 010.	
**010**	Soft packed, slightly mottled reddish brown to dark reddish brown (5YR 3/3, 4/3) gravelly silt with calcareous ash inclusions. All finds decrease in density compared to the contexts above. Most of the finds are bones from large mammals. Since this context was thicker than 10 cm, it was divided into 10 cm subsections (010A, 010B)	Layer 7
**011**	Localized occurrence on the southern edge of K13 and L13. Loose pinkish grey to reddish grey (5YR 6/2, 5/2) dry silt with small amounts of carbonate nodules and roots. Much less gravelly than Context 010, with slightly more frequent shells and lithics. A roughly circular concentration of carbonate nodules, may be a dripline lag (from rainwater run-off from the roof).	Layer 8

The visual assessment of find density distribution reveals varying artifact density across the stratigraphy, with clear density hotspots (Fig 5). When viewing the hotspots by material type, there is a clear difference between the upper contexts that bear ceramic finds (001–003) and the aceramic contexts below (005–011), with a higher concentration of lithics. The thin Context 004 (Layer 3) separates the two. Context 005 is characterized by the high frequency of marine shell (including the oyster *Saccostrea cucullata*, of intertidal/mangrove habitat), indicating the inclusion of marine resources in the foraging economy of the PYS occupants. From Context 007 (Layer 5) to the base of the excavation (Context 011/Layer 8), bone and lithics are the dominant find classes, although marine shells are occasionally present.

**Fig 6:**
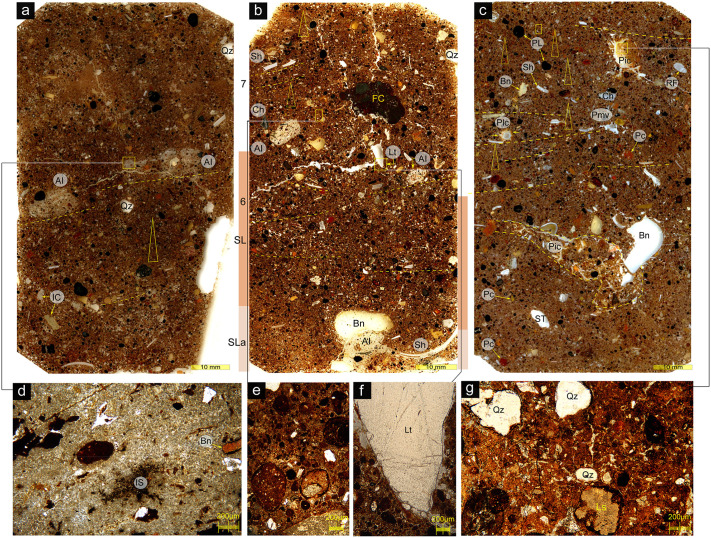
Composition and structure of correlative deposits from Trench 4 (for their spatial relationship with the 2020 excavation, see Fig 2). a–c: Flatbed scans of uncovered thin sections; d–g: photomicrographs (PPL). a) Transition between Layer 8 (correlative with Context 011: the base of the 2020 excavation) and the underlying Layer 9 (not excavated in 2020). Yellow triangle indicates fining-up domains. In Trench 4, the line of ash intraclasts (AI) and clusters of closely-packed coarse particles at mid-section (yellow lines) could be traced laterally to ash deposits and coarse limestone clasts (spall) at the top of Layer 9. b) Boundary between coarser Layer 6 (Context 090) above and Layer 7 (Context 010) – finer-grained sandy loam (SL) and even finer sandy loam with reworked ash (SLa) – below. At the lower part of the section (Layer 7: SLa), a large intraclast of laminated ash (AI) with inclusions of charcoal (black) and heat-reddened sediment (rip-up clasts: reddish-brown) adheres on a large bone fragment (Bn). The linear arrangement of (locally imbricated) platy and ovoid particles (yellow lines) and the fining-up domains (yellow triangles) are interpreted as primary depositional structures. The rounded ash intraclasts (AI) at mid-section were cemented, possibly due to exposure on the cave floor, before their erosion and redeposition, presumably from further upslope. Lt, an angular, pointy fragment of coarse-crystalline quartz, is interpreted as stone-knapping refuse. c) Transition between Layer 5 (Context 007) above and Layer 6 (Context 008) below. Note the poor sorting, linear arrangement of many platy particles (yellow lines), and fining-up domains in parts of the section (yellow triangles). Pores Pic (the larger one highlighted) are bioturbation channels filled with illuvium (see Fig 6g). The lining of pore Plc (arrows) is compositionally and microstructurally similar to that of Pic and is therefore also intepreted as illuvial in origin. Pore Pmv has been deformed by wetting and drying, but lacks illuvial filling. d) Ash intraclast at the boundary between layers 8 and 9: laminated, partly phosphatised and wood ash with inclusions of burned bone (Bn) and mineral grains (mainly quartz and ferricrete), and dark organic or organomineral (iron-manganese?) impregnation spots (Is). (Arrows: recrystallisation of ash to clear calcite microspar) e) Fining-up domain in Layer 6: Well-rounded coarser particles (bedrock limestone, intraclasts, pisolites) set in a poorly-sorted finer matrix (porphyric c/f-related distribution). The coarser particles become progressively smaller, less frequent, and more openly-spaced up-section. f) Detail of inferred stone-knapping refuse in Layer 6. g) Very poorly-sorted fill of bioturbation channel in Layer 5: grains of quartz (Qz), limestone (LS) and ferricrete ‘float’ in a matrix of ferruginised silty clay. The channel fill resulted from illuvial deposition by relatively high-energy percolating water, and may include particles translocated from overlying horizons.*Key:* PL: iron (+manganese?) pisolite; FC: ferricrete; PC: pedoclast (mainly red clay with quartz silt); Qz: quartz; IC: intraclast; RF: rock fragment (cave-wall limestone); LS: limestone; ST: speleothem; Lt: stone-knapping refuse; Bn: bone; Sh: shell; Ch: charcoal; IS: impregnation spot; P: pore (ic: infilled channel; lc: lined channel; mv: multiconcave vugh).

Contexts 001–003, with large quantities of local Tana Tradition ceramics, date to the late Holocene (late first–early second millennium CE) [48]. These contexts likely correlate to Layers 1–2 from the previous excavations [10]. Context 004, beginning 10–15 cm below the modern surface, marks a transition from ceramic (above) to aceramic deposits below. This thin deposit was identified based on the sudden decrease in find density and the presence of four large (10s of cm long) limestone blocks sitting along a horizontal plane that may represent a palaeofloor. Below this, ceramic fragments virtually disappear, lithic density increases, and large mammal bones and teeth appear. This deposit may represent the northward extension of Layer 3 in earlier excavations. The aceramic–ceramic transition appears to have taken place during the deposition of this context. Context 005, with large quantities of marine shell, is likely equivalent to Layers 4 and 5 in previous excavations [49]. A dense accumulation of lithics recovered on a horizontal plane, and a core-blade refit identified during excavation hint at the improving stratigraphic integrity at this depth (S1 Video), though future research could examine the vertical and horizontal conjoining line of refits from this layer [as in [50]] to further test the stratigraphic integrity of this context at PYS. Immediately below this, Context 007 appears to correlate with the basal part of Layer 5 in previous excavations. Correlative deposits from Trench 4, closer to the cave wall (S1 Table, PYS 5/6), contain abundant microscopic fragments of food remains, charcoal, and tool-knapping refuse set in a matrix of sandy silt (Fig 5). These were interpreted as human occupation debris reworked on the cave floor, perhaps from dense sheetflow/sheetwash. The preservation of coherent ash intraclasts within these may suggest that hearths or hearth rake-out deposits were exposed, and partly cemented on the cave floor for some time before their reworking. Context 007 grades gradually into Context 008, correlative with Layer 6 in previous excavations. This thin deposit fines upwards from gravely silt (at its base) to silt; compared with its overlying context, it contains fewer snails and no marine shells. Correlative near-wall deposits in Trench 4 (S1 Table) are compositionally similar to the overlying deposits, with subtle microstratigraphic transitions across mm to cm (Fig 6). Human occupation debris was mixed with sediment inputs from outside the cave, as indicated by the presence of variable pedoclasts (reworked soil fragments: mainly ferruginous silty clay with fine quartz silt, and occasional fragments of limpid clay coatings). Some of the pedoclasts could have been introduced inadvertently by people, on items of dressing, feet, foodstuffs, etc. Algal speleothem fragments in the deposit suggest that sediment sources included unroofed parts of the cave, exposed to light—presumably the walls of the already unroofed chamber. Deposition probably took place by weakening sheetflow on the cave floor, as suggested by poor sorting, horizontal particle orientation and fining-up depositional domains. A subtle microstratigraphic boundary with a higher concentration of bone, shell, burnt residues, and knapping flakes may indicate the formation of a floor between episodes of rain-triggered sheetwash. Postdepositional bioturbation by arthropods and plant roots was extensive, but vestiges of the original depositional structure (e.g., fining-up cycles, imbrication) were preserved.

Context 011 occurs only locally in the 2020 trench (Fig 6, Table 1), but appears to correlate with the much thicker Layer 8 in earlier excavations. It consists of loose, dry gravely silt with small amounts of carbonate nodules and roots. A lower gravel content and slightly more frequent shells and lithics distinguish this from overlying Context 010. Correlative Layer 8 deposits from Trench 4 (Fig 6) contain abundant human occupation debris (food remains, including burnt bone fragments, ash and charcoal, amorphous charred organics, heat-reddened intraclasts) deposited on a well preserved palaeofloor, with fire-reddened domains probably marking the substratum of in situ hearths. Burning residue include both woody charcoal and (mainly) charred non-woody tissue. Unlike in later (early Holocene) hunter-gatherer sites in eastern Africa (e.g., Hora 1, Malawi: [51]), no evidence for the utilisation of deadwood as fuel (e.g., remnants of termite galleries in ash) was found at PYS. As with overlying deposits, human occupation residue was mixed with sediment from elsewhere within and outside the cave, as indicated by the frequent and diverse intraclasts and pedoclasts. Postdepositional alteration included cementation by dripwater, ash recrystallisation, and phosphatisation of carbonate particles (mainly shell and ash). Distinct phosphatisation horizons may have developed under palaeofloors.

A significant stratigraphic transition was identified between contexts 008 and 010. The contact between these is marked by a layer of loosely packed silt with gravel, which can be traced laterally around the western edge of the existing trench, where it slopes downward significantly towards the southern end of the trench. The 008/010 context boundary is correlated with the sharp boundary between layers 6 and 7, which, in previous excavations (closer to the cave wall), signaled a marked increase in the density of colluvial input and burning residues [10]. This sloping boundary marks the top of several southward-dipping stratigraphic units, previously observed in the western wall of the main excavation area, and probably forming part of a talus fed by colluviation from the overcave surface. Correlative near-wall deposits from Trench 4 were interpreted as floor colluvium deposited above an activity surface (possibly a lateral correlative of the gravely 008/010 boundary). The sharp increase in putative human inputs from Layer 6 up probably indicates intensification of human activity at and/or upslope of the trench site. Lithics per liter per year confirms that Layer 6 represents a peak in human occupation intensity (S1 Fig.). Bioturbation has obscured primary stratification, but fining-up domains may indicate deposition from sheetflows over the sloping surface. Deeper in Layer 6, partly conjoinable ash intraclasts and rubified particles may have resulted from the reworking of hearth waste towards the cave wall on the inclined cave floor. These reworked ash layers are interlayered with sandy silty colluvium with abundant human habitation debris. Fragments of delicate sediment crusts indicates episodic reworking of cave floor(s) into this colluvium. The high quartz content and diverse silicate minerals in the colluvial matrix may signal increased sediment influx from outside the cave and, possibly, reduced rates of mineral weathering (hydrolysis). This may be consistent with a drier regional climate and sparser vegetation in the sediment source (which, if the aeolian processes contributed to deposition, may have extended beyond the overcave surafce-e.g., to the coastal plain). Humans may have also introduced sediment from various sources inadvertently (e.g., on items of dresing, feet, foodstuffs, etc.).

### Postdepositional change

Pervasive bioturbation by arthropods and tree roots is evident both macroscopically (blurring of stratigraphic boundaries, cm-scale macroburrows, ant and termite galleries), and microscopically, in laterally correlative deposits of the 2013 excavation (channels and chambers, granular/crumb microstructure locally, fecal pellets of indeterminate origin: S1 Table, Fig 6). Evidently, these deposits underwent pedogenesis in tandem with their deposition, as is the case in many caves in the tropics [48,49]. The presence of conjoinable ceramics and lithics in some of the 2020 contexts, the broadly consistent increase in radiocarbon age down-profile (with only few reversals), and the preservation of some of the original structure (e.g., successive fining-up domains, linear arrangement and local imbrication of elongate/platy particles (Fig 6), fragmentary but traceable surfaces in laterally correlative layers (S1 Table) indicate that bioturbation did not obliterate the original stratification by completely homogenizing the deposits. In these conditions, small particles (< 1–2 cm) may have moved across the stratigraphy (perhaps more so in the more exposed and bioturbated setting of the 2020 trench), while larger ones (> 2–3 cm) may have remained largely in place.

No guano deposits were identified in the excavation of this relatively exposed part of the cave. In keeping with this, phosphatization is patchy, and often associated with textural microstratigraphic boundaries of reduced porosity, accumulation of fine sediments, as well as ash and charred organics, interpreted as probable palaeosurfaces (S1 Table, Fig 6). At places, phosphates replace shell and speleothem fragments (although the latter may have been phosphatized before their fragmentation and deposition on the cave floor). It is unknown whether phosphate deposition was driven by ave fauna that may have occupied this part of the cave between periods of human presence, or by human waste. Calcite deposition and recrystallisation of ash to clear calcite microspar are also patchy. Fragments of hardened cave ground evidence the formation of stable floors (perhaps, under drip points and/or the dripline), and their subsequent disruption. Although iron (+manganese?) nodules are frequent in the PYS deposits (Fig 6), most were probably transported from the lateritic soil above the cave where such nodules abound in karstic cavities. Orthic (*in situ*) iron oxide nodules, resulting from seasonal waterlogging (probably favored by the high clay content of the PYS deposits) are few and mainly incipient. Deposits in the upper part of the PYS profile, therefore, appear to have remained in consistently vadose conditions (above the groundwater table) since their deposition.

### Radiocarbon chronology

A total of 16 new radiocarbon dates from the PYS excavations are presented here (Table 2, S2 Table). These newly reported ages comprise twelve samples from 2020, all of which were piece-plotted *in situ* during the excavation. Four additional samples from earlier excavations were also tested for this study, to fill gaps in the sequence and/or test correlations with earlier established layers: A charcoal sample from Trench 6 (Context 609) was dated, as no suitable material for dating this context was recovered from the 2020 excavation. Likewise, a charcoal sample from a hearth cut into Layer 3 in Trench 7 (Context 706) was dated to obtain a new age for this layer. Two marine shell samples (*Saccostrea cucullata*) from layers 4 and 5 (2013) were dated alongside *S. cucullata* from Context 005 of the 2020 excavation to test layer–context correlation.

**Table 2:** Radiocarbon ages from the 2013, 2017 and 2020 excavations at PYS.

LabNo	Context	Layer	Sample Type	Conventional radiocarbon age (^14^C years ± 1σ)
ETH-129776	002	1	Charcoal	640 ± 21
GU56785/ SUERC-96613	003	Fill	Charcoal	2256 ± 23
ETH-129775	003	Fill	Charcoal	2532 ± 22
ETH-129774	004	3	Charcoal	2498 ± 21
GSU55615/ SUERC-94580	706	3	Charcoal	2907 ± 31
GU62459/ SUERC-107525	408B	4	*Saccostrea cucullata*	5278 ± 24
GU56786/ SUERC-96617	005A	4/5	Charcoal	4515 ± 23
GU56788/ SUERC-96618	005D	4/5	Charcoal	4527 ± 25
GU62462/ SUERC-107531	005A	4/5	*Saccostrea cucullata*	5922 ± 24
GU62461/ SUERC-107530	005A	4/5	*Saccostrea cucullata*	7661 ± 24
GU56790/ SUERC-96619	007	5	Charcoal	5820 ± 24
GU62460/ SUERC-107526	408C	5	*Saccostrea cucullata*	7633 ± 24
GU55620/ SUERC-94588	609	6	Charcoal	13690 ± 31
GU56787/ SUERC-0	010A	7	Bone	No date
GU56789/ SUERC-0	010A	7	Bone	No date
GU56791/ SUERC-96620	011	8	OES bead	21139 ± 61

**Fig 7:**
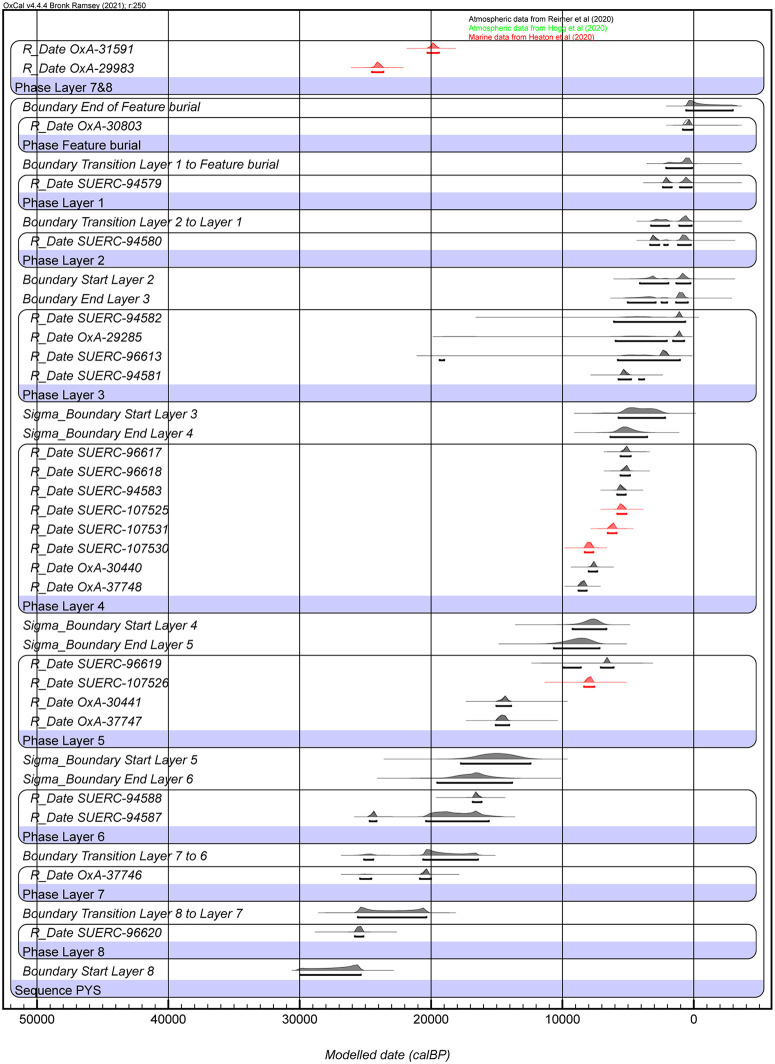
Bayesian modelled ages for PYS Layers 1-8, and transitions. See S1 File for OxCal code.

We combine these new radiocarbon ages with 9 previously published ages from this upper part of the sequence, to produce a new Bayesian model for PYS (S1 File and S2 Table). The new Bayesian model for the upper part of the sequence begins with the burial from the mid-second millennium AD [31] (Fig 7). Layers 1 and 2 correspond to the early and later parts of the 1^st^ millennium AD respectively. Layer 3 has a broader span of 4.6–2.4 ka. The dates of 2.5 and 3 ka from contexts 004 and 706, respectively, testify to a previously unknown occupation phase at PYS, between the mid-Holocene and the Middle Iron Age. Layer 4 is mid-Holocene (7.8–5.3 ka). Layer 5 may span an occupation hiatus: our new Bayesian model places it in the mid-Holocene (8.5–6.6 ka), but terminal Pleistocene dates (15 ka) were previously reported from correlative deposits closer to the cave wall, with Layer 6 also being terminal Pleistocene (16.6–15 ka). Layer 7 dates from the terminal Pleistocene to the Last Glacial Maximum (LGM) (22.9 ka). Layer 8 represents the earlier part of the LGM (27.6–22.9 ka).

## Discussion

### Sediment depth and archaeological potential

Overall, the PYS sediment fill registers a long period of episodic deposition and varying intensity of human occupation in a cave chamber that was itself being reshaped by episodic unroofing. The 3.5 m of cave fill excavated over successive seasons indicates that colluviation from outside and within the cave (further upslope from the trench site), roof and wall disintegration, and human occupation (that mainly yielded burning byproducts, food remains, and lithic knapping refuse) were the main processes of sediment deposition in the excavated part of the cave. These processes interacted variably across time to produce a discontinuous and variegated sediment record. The depth and temporal range of this record below the present cave floor remain unknown. Multiple lines of geological and geomorphological evidence (multilevel cave morphology, vertical development of chambers, half-buried stalagmites whose base is at some depth under the cave floor, regional depth of karstification) indicate that hypogenic speleogenesis generated large, deep karstic voids that began to receive sediment as the cave became exhumed and its celling was breeched. At PYS, sediment depth exceeds the limits of the current excavations and probing (3.5 and 5 m respectively), and may extend well into MIS 5 (if not much earlier).

Extrapolating from the exposed chamber morphology, sediment thickness can be expected to be greater in the central part of the chambers and/or in feeders (hypogenic cavities of vertical morphology). Notwithstanding this, we infer that the parts of the cave with the strongest archaeological potential are along the side of the chambers, near the cave wall, and within enclosed chambers, where the overhang makes for a more sheltered setting. A similar situation can be found at nearby Panga ya Mwandzumari, where excavation in the center of an open-roofed chamber produced no archaeological remains, while excavation under a rockshelter dripline produced a rich sequence [17]. Driplines thus form key hydrological, sedimentological, ecological, and experiential boundaries that partition human inhabitation across the large cave space. Dripline processes also result in the formation of distinct deposits that could be recognizable in the stratigraphic record: At points of recurrent drip (e.g., along the drip line or under active stalactites), water washes away the finer sediment matrix thus concentrating coarser particles, including bone and articles of material culture from different periods, into drip lags. These palimpsest accumulations are often encrusted with calcium carbonate. Such features have not yet been conclusively identified in the PYS excavations, concurring with above-ground observations that we are still inside the dripline.

### Human activity, sediment deposition, and the evolution of the cave floor

**Fig 8:**
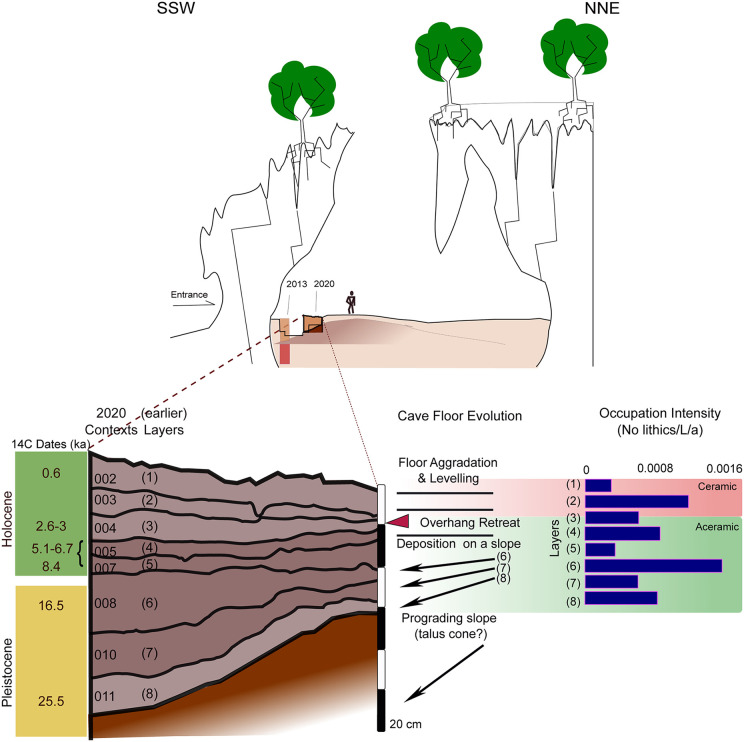
Relationship between the evolving cave chamber and floor morphology, sediment architecture and occupation intensity at PYS over the last ca. 25.5 ka.

By extending the exposed stratigraphy to the north of the contiguous trenches 1, 3, and 4 and toward the modern dripline (Figs 1 and 2), the 2020 excavation gave us a better understanding of the links between human inhabitation and the evolution of cave floor and walls at PYS since the terminal Pleistocene. The 2020 sequence appears to have a thicker Holocene accumulation than the previous excavations, with a more abrupt transition to the Pleistocene (though no dates were obtained from the probable terminal Pleistocene Context 008 (Layer 6) in this excavation). The west section of the main trench testifies to the dominance of colluvial inwash, in the lower, terminal Pleistocene sections of the trench, which dip clearly to the south (Figs 6 and 8), from the cave floor to the north of the trench. This depositional architecture is attributed to a colluvial cone formed by sediment input from the overcave surface into the center of the chamber. Sediment deposition accelerated in terminal Pleistocene times, evening the morphology of the cave floor. Lithic density and micromorphological evidence from correlative deposits suggest that these higher rates of sediment deposition were to a large extent mediated by human presence in the cave (Fig 8). Sediment deposition was episodic, punctuated by periods of floor stabilization (with the development of palaeofloors in the contiguous Trench 4), limited human presence or abandonment (when colluvial inwash was the dominant material deposited), and, possibly, one episode of overhang retreat in mid-Holocene times.

Consistent with previous seasons, decorated sherds are of the Tana Tradition, which in this region ranges in age from 1250 to 850 BP [48], were identified in the upper, ceramic-bearing contexts of PYS (001–003), Ceramic fragments were unearthed lying flat, suggesting that they were deposited onto a horizontal plane (similar to that of late Holocene ceramic deposits in the cave: Fig 5), with minimal postdepositional disturbance. Previous studies both at PYS [17,18] and in the broader study region (e.g., [52,53]] show the presence of crops in contexts with Early Tana Tradition pottery, and have firmly established the arrival of a broad suite of pan-African crops to the east coast of Africa by the 7th century CE. Direct dating of botanical remains from PYS [51] suggests that the presence of crops in the aceramic deposits is the result of downward translocation through the soft, loose sediment. The absence of sharp stratigraphic boundaries and the evidence for bioturbation by plant roots and soil fauna further indicate that charcoal and other small botanical remains may be reworked vertically in the upper 20–30 cm of stratigraphy. In addition, the proximity of the 2020 trench to the dripline may have resulted in the formation of drip lags where coarser particles of various age were juxtaposed in a palimpsest (as elsewhere in PYS).

At ~30 cm below surface, in aceramic Context 005, the dense deposition of lithics on a flat plane hints that a palaeofloor was preserved to some degree. Future research could examine the vertical and horizontal conjoining line of lithic refits from this layer [as in [50]] to further test the stratigraphic integrity of this context. Micromorphological evidence from deposits correlative with contexts 006 and below, about 2.5 m to the south of the 2020 excavation also indicates that bioturbation, although pervasive, did not completely efface the original stratigraphy (S1 Table). In these conditions, smaller particles likely moved down-profile but larger ones may have mostly remained in place.

Approximately 60–70 cm below the surface, at the context 008/010 boundary, coarser inclusions and silt on a sloping surface suggest episodic colluvial deposition. This is accompanied by a marked decrease in artifactual finds, and may represent the terminal phase of a period of more limited human activity in this part of the cave, when most of the sediment originated from extra-cave colluvial inputs.

The upper 80 cm of the stratigraphy contains two breaks in occupation. The first is the presence of roof fall in Context 004 that divides the ceramic and aceramic layers. Dates from directly above and below this context indicate an over 2,000-year gap, with the upper occupation phase now found to begin prior to the Iron Age, and the phase below, characterized by the strong contribution of aquatic molluscs in the diet, relating to the mid-Holocene sea-level highstand [49].

The second break in occupation reflects the LGM to terminal Pleistocene transition. Contexts 001–008 share rich and well-preserved macrobotanical finds with radiocarbon dates from the terminal Pleistocene and Holocene. However, charcoal and other organic finds decrease markedly in contexts 010 and 011. A large mammal bone from Context 010 failed to yield an age, while the underlying Context 011 yielded an ostrich eggshell bead directly dated to 25 ka. A radiocarbon date from the equivalent of Context 010 in Trench 6 (Layer 7) produced an age ca. 16,550 cal BP. The profile of the combined Trench 4, 5, and 7 excavations indicates that the sloping morphology of the cave floor was leveled by Layer 6 in the terminal Pleistocene. It is possible that increased vegetation on the overcave surface after the LGM reduced colluvial input to the cave. Debris from intensive human occupation at that time, alongside more limited colluvial sediment, gave the cave floor its present, horizontal morphology (Fig 8).

Much remains to be learned about the interplay between cave morphology evolution and the spatial patterning of human habitation in PYS over the last > 78,000 years. As our composite stratigraphies and observations nevertheless show, the configuration of the cave has changed remarkably since the Late Pleistocene (ca. 24,500 BP), and this change has affected the distribution of human activity in the cave space. The leveling of the slope towards the cave wall due to cave floor aggradation in the terminal Pleistocene, for instance, may have influenced the range of human activities taking place there, and thus the archaeological assemblage preserved in this part of the cave. The location of main occupation spots further up this slope, closer to a dripline that extended further out towards the chamber center, could explain the mixed composition of microscopic habitation debris as refuse discarded or colluviated down slope towards the cave wall. Erosion of this slope could also explain radiocarbon age reversals and the juxtaposition of earlier and younger artifacts and ecofacts in colluvial downslope deposits (as inferred for cave-entrance facies in Kuumbi Cave in Unguja (Zanzibar) [47]). It is possible that earlier shifts in the intensity of human occupation (e.g., the marked increase around 60,000 BP, recorded in previous excavations (layers 14–13)) in this part of the cave [10] were also conditioned by reconfiguration of the cave space (e.g., ceiling collapse and the transformation of a dark recess into a more open chamber). Evidence for this (e.g., ceiling collapse breccia) could be sought to the north of the 2020 trench and at greater depths.

## Conclusion

Panga ya Saidi contains an archaeological record spanning at least the last 78,000 years, and provides a much needed near-coastal counterpoint to the well-known inland records from eastern Africa. Controlled excavation in PYS has reached ~3.5 m below the cave surface. The abundant and coalescent cupolas formed due to an earlier, hypogenic phase of cave formation conditioned ceiling collapse as the cave became exhumed during the Quaternary. This diachronous process (which is still ongoing) dramatically re-modified the cave space and created variegated morphologies and microhabitats, such as rockshelters, unroofed chambers, and dark alcoves. Sediment deposition, predominantly as colluvial inwash from the over-cave surface, interspersed with human occupation debris and roof fall, generated a sediment archive at least 5.5 m thick (according to excavation and probing), and perhaps considerably thicker in the center of the cave chambers (when considering the depth of karstification in the area and the 3D morphology of the cave chambers).

Human inhabitation was spatially patterned within this highly differentiated cave environment. Dark and enclosed chambers were used as ancestral burial sites within living memory, and access to these continues to be regulated by ritual. The most frequent, iterative human use of PYS appears to have concentrated at the sides of accessible chambers, in rockshelter-like embayments under bedrock overhangs that offer more protection from the elements while still providing natural light. We infer that these zones of the cave, from the dripline to the cave wall, are the most promising archaeologically, though dark caverns are also likely to show more limited use. The main excavation trenches at PYS are located in one of these key sheltered areas: behind a high-roofed dripline in the southern chamber. More exposed parts in the center of unroofed chambers may contain the deepest stratigraphy, but their archaeological potential appears to be more limited, perhaps due to the lack of shelter from heavier rain and denser vegetation (which may also account for the heavier bioturbation of deposits there, as compared with the chamber margins). This estimate, nevertheless, should be qualified by the recognition that this differentiated cave environment has evolved morphologically throughout the Late Quaternary.

Differentiation of human activity across the evolving cave space manifests at various scales: The lower artefact density of the pre-ceramic deposits in the more exposed, northern part of the 2020 excavation stands in contrast to the higher artifact density in the more sheltered, southern part. This may reflect more regular occupation of the inner, sheltered part of the rockshelter. Alternatively, it may have resulted from the accumulation of human occupation debris against the cave wall, at the end of cave-floor transport gradients: these gradients may have been behavioral (e.g., iterative sweeping, rake out, floor cleaning, etc.) and/or hydrological-sedimentological (e.g., water washing in from the overhang). Excavation along these inferred gradients (that is across the dripline) and more detailed sedimentological (including micromorphological) and taphonomic work is required to reconstruct terminal Pleistocene–Holocene site-formation processes in this part of PYS.

Bioturbation and human activity (e.g., cut-and-fill structures) in the uppermost 20–30 cm of the stratigraphy suggest that a more secure determination of the age of small finds may require direct radiocarbon dating of individual pieces. With this approach, the 2020 excavation revealed a previously undocumented 2.5 ka occupation phase at PYS, between the known mid-Holocene and Middle Iron Age (Tana) occupations. The presence of larger clasts in this otherwise thin layer likely indicates the development of a stable floor on which pottery and preceramic artefacts and ecofacts accumulated diachronously, as a palimpsest.

Below 30 cm, stratigraphic integrity improves. Finds from the mid and lower levels of the 2020 excavation show some horizontal deposition of artifacts and ecofacts that may reflect past surfaces. Despite some degree of downward intrusion of small (<cm) particles (e.g., crop seeds), the majority of finds appear largely *in situ*, with no macroscopic signs of taphonomic disturbance.

The transition between contexts 008 and 010 marks a major shift in floor morphology and depositional regime. The southward (toward the wall)-dipping slope–probably the flank of a colluvial cone that developed since at least early MIS2 was levelled by onlapping sediment in terminal Pleistocene times. 3D find plots and overall artifact deposition rates, in particular lithics, suggest an increase in occupation intensity at the time of this transition, with anthropogenic input perhaps becoming the dominant sediment source. From this time onwards, deposition took place on a more or less horizontal aggrading floor, resulting in horizontally bedded deposits. This increased contribution of human occupation debris to sediment deposition and the filling up of the space from the slope to the wall probably occurred during the deposition of Context 008 (~Layer 6), bracketed by the 20 ka date for Layer 7 [13] and the 14.5 ka date for Layer 5. The new date for Layer 6 from Trench 6 suggests that this layer formed 16.5 ka. The question remains as to when the slope initially formed, and the nature of its underlying deposits.

PYS, with a record of occupation over the last 78,000 years, stands as a pivotal archaeological site for understanding *Homo sapiens* behavior in eastern Africa. Our new investigations, including digital recording and 3D scanning, illuminate the intricate interplay between the changing cave morphology, patterns of sediment deposition, and spatially differentiated human activity within this unique cave environment. The findings underscore the significance of this site for understanding cultural transformations during the Late Pleistocene and Holocene. The potential for future research remains high. As we delve deeper into the stratigraphic complexities of PYS, we anticipate that these insights will ultimately enrich our understanding of human evolution and adaptation in the region, reinforcing its critical role in the broader archaeological narrative of the deep-time history of our species.

## Supporting information

S1 File OxCal code used to produce Bayesian model of ages at PYS. (DOCX)

S1 Table Thin-section micromorphology of deposits from the 2013 excavation (Trench 4; samples M16–M13) correlative with contexts 2020 contexts. (DOCX)

S2 Table Excel table of modelled and unmodelled results for 25 radiocarbon samples. 68% and 95% confidence intervals.
(XLSX)

S1 Video A core and flake refit, identified during excavation within a dense debitage cluster. The proximity of these artifacts to one another supports a degree of stratigraphic integrity, and minimal disturbance by post depositional factors in Context 005.
(MP4)

S1 Fig Lithics per liter per year for Layers 1–8 at PYS. (DOCX)
