# Supplementary material for: Cave morphology and human-mediated sediment deposition: Late Pleistocene to Holocene evolution of the cave floor at Panga ya Saidi, coastal Kenya
Source: PLoS One. 2026 May 20;21(5):e0347491. doi: 10.1371/journal.pone.0347491 (PMC13189332; doi:10.1371/journal.pone.0347491)
Supplement: S1 File — (DOCX) [file pone.0347491.s001.docx]

**S1 File. OxCal code used to produce Bayesian model of ages at PYS.**

Options()

{

Resolution = 250;

};

Plot()

{

Outlier_Model('General',T(5),U(0,4),'t');

Curve('IntCal20','IntCal20.14c');

Curve('SHCal20','SHCal20.14c');

Mix_Curves('Mixed','IntCal20','SHCal20',U(0,100));

Curve('Marine20','Marine20.14c');

Sequence('PYS')

{

Boundary('Start Layer 8');

Phase('Layer 8')

{

Curve('=Mixed');

R_Date('SUERC-96620', 21139, 61){Outlier('General', 0.05);};

};

Boundary('Transition Layer 8 to Layer 7');

Phase('Layer 7')

{

Curve('=Mixed');

R_Date('OxA-37746', 16910, 65){Outlier('General', 0.05);};

};

Boundary('Transition Layer 7 to 6');

Phase('Layer 6')

{

Curve('=Mixed');

R_Date('SUERC-94587', 20360, 48){Outlier('General', 0.05);};

R_Date('SUERC-94588', 13690, 31){Outlier('General', 0.05);};

};

Sigma_Boundary('End Layer 6');

Sigma_Boundary('Start Layer 5');

Phase('Layer 5')

{

Curve('=Mixed');

/*

Charred seed sample deemed to be an outlier (repeated measurements available: OxA-26775: 522 ±25 yrs BP; OxA-26776: 536 ±24 yrs BP;). Combined result shown below for reference.

R_Date('Combined 103E OxA-26776 & OxA-26775', 529, 18){Outlier('General', 0.05);};

*/

R_Date('OxA-37747', 12435, 50){Outlier('General', 0.05);};

R_Date('OxA-30441', 12375, 50){Outlier('General', 0.05);};

Curve('=Marine20');

Delta_R('Local marine 5',-45,44);

R_Date('SUERC-107526', 7633, 24){Outlier('General', 0.05);};

Curve('=Mixed');

R_Date('SUERC-96619', 5820, 24){Outlier('General', 0.05);};

};

Sigma_Boundary('End Layer 5');

Sigma_Boundary('Start Layer 4');

Phase('Layer 4')

{

Curve('=Mixed');

R_Date('OxA-37748', 7692, 34){Outlier('General', 0.05);};

R_Date('OxA-30440', 6797, 36){Outlier('General', 0.05);};

Curve('=Marine20');

Delta_R('Local marine 4',-45,44);

R_Date('SUERC-107530', 7661, 24){Outlier('General', 0.05);};

R_Date('SUERC-107531', 5922, 24){Outlier('General', 0.05);};

R_Date('SUERC-107525', 5278, 24){Outlier('General', 0.05);};

Curve('=Mixed');

R_Date('SUERC-94583', 4792, 31){Outlier('General', 0.05);};

R_Date('SUERC-96618', 4527, 25){Outlier('General', 0.05);};

R_Date('SUERC-96617', 4515, 23){Outlier('General', 0.05);};

};

Sigma_Boundary('End Layer 4');

Sigma_Boundary('Start Layer 3');

Phase('Layer 3')

{

Curve('=Mixed');

R_Date('SUERC-94581', 4603, 31){Outlier('General', 0.05);};

R_Date('SUERC-96613', 2256, 23){Outlier('General', 0.05);};

R_Date('OxA-29285', 1212, 23){Outlier('General', 0.05);};

R_Date('SUERC-94582', 1189, 31){Outlier('General', 0.05);};

};

Boundary('End Layer 3');

Boundary('Start Layer 2');

Phase('Layer 2')

{

Curve('=Mixed');

R_Date('SUERC-94580', 2907, 31){Outlier('General', 0.05);};

};

Boundary('Transition Layer 2 to Layer 1');

Phase('Layer 1')

{

Curve('=Mixed');

R_Date('SUERC-94579', 2098, 31){Outlier('General', 0.05);};

};

Boundary('Transition Layer 1 to Feature burial');

Phase('Feature burial')

{

Curve('=Mixed');

R_Date('OxA-30803', 388, 27){Outlier('General', 0.05);};

};

Boundary('End of Feature burial');

};

Boundary('=Start Layer 8');

Phase('Layer 7&8')

{

R_Date('OxA-29983', 20835, 75){Outlier('General', 0.05);};

R_Date('OxA-31591', 17170, 75){Outlier('General', 0.05);};

};

Boundary('=Transition Layer 7 to 6');

};
