# Supplementary material for: Cave morphology and human-mediated sediment deposition: Late Pleistocene to Holocene evolution of the cave floor at Panga ya Saidi, coastal Kenya
Source: PLoS One. 2026 May 20;21(5):e0347491. doi: 10.1371/journal.pone.0347491 (PMC13189332; doi:10.1371/journal.pone.0347491)
Supplement: S1 Table — (DOCX) [file pone.0347491.s002.docx]

**S1 Table.** **Thin-section micromorphology of deposits from the 2013 excavation (Trench 4;** **samples M16–M13) correlative with contexts 2020 contexts.** Here, we report the results of micromorphological analyses for, correlative with parts of the 2020 stratigraphy (base of Context 007 to Context 011, and the very top of the underlying, southward-dipping sediments) Notes on terminology and abbreviation: ‘Coarse’ particles (mineral/biogenic): maximum size ≥ 50 μm; ‘Fine’ particles (mineral/biogenic): maximum size < 50 μm; MS: maximum particle size in the unit (measured); Particle size classes: G: gravel-sized ( ≥ 2000 μm); cS: coarse-sand-sized (2000–600 μm) ; mS: medium-sand-sized (600–200 μm); fS: fine-sand-sized (200–50 μm); s: silt-sized (50–5 μm); c: clay-sized (< 5 μm). CFRD: Coarse-to-fine-related distribution—a measure of the spatial relationship between coarse and fine particles in the deposit. Observation conditions: PPL: plain polarised light; XPL: cross-polarised light; OIL: oblique incident light. MSB: microstratigraphic boundaries, broadly correlative with macrostratigraphic (layer) boundaries in the 2013 stratigraphy.

| **M16 (PYS 5/6**): **Layer 5/6 boundary**, potentially correlative with the boundary between contexts **007** and **008** in 2020. |
| --- |
| **Basal Layer 5**, correlative with basal **007**: *Poorly sorted, heterogeneous beige-brown sandy loam with iron pisoliths, bone, and shell fragments.* Separated from the underlying unit by **(MSB1**)—a subtle textural boundary *of lower porosity and smaller grain size*. |
| **FABRIC Microstructure:** Massive at the cm–mm scale, disrupted by channels and chambers. Parallel lamination and imbrication of platy particles at the base (immediately above MSB1), followed by a fining-up trend. **Porosity:** 5–10%. Pores as in PYS 6 (upper). **CFRD:** Open to close porphyric at various scales; also chitonic locally.    **GROUNDMASS** As in PYS 6 (upper), but somewhat less rubified.    **MINERAL CONTENT COARSE:** Overall as in PYS6 (upper). **Quartz:** 10–15% (MS: 2240 μm); **Fe-Mn pisoliths/ferricrete:** ~25–30% (MS: 4640 μm). **Limestone & speleothem:** Rare (≤ 2%); probably less frequent than in PYS 6 (upper). **FINE:** As in PYS 6 (upper); **Quartz silt:** 2–4%.    **BIOGENIC CONTENT COARSE:** Overall as in PYS 6 (upper), but faunal remains are less frequent and smaller. **Bone:** 2–5%; **Shell**: 5–6%; **Charcoal:** 2–7% (upper layer). **Ash:** ~2%. **Artefacts:** One possible quartz flake. **FINE:** As in PYS 6 (upper). **Carbonised organic silt**: 5–7%.    **PEDOFEATURES**As in PYS 6 (upper) and the two underlying two contexts. Diverse pedocalsts (soil fragments). |
| **INTERPETATION** Redeposition of human occupation debris (food remains, fuel and tool-knapping refuse) by cave floor processes, perhaps at some distance from foci of human activity (hearths, cooking sites, rake out)? Horizontal particle orientation and fining-up depositional cycles above MSB1 may indicate deposition by weakening flow (consistent with sheetwash). Ash intraclasts may indicate that hearths (or hearth rake-out deposits) were exposed for some time, and partially cemented, before their disruption and reworking. Human occupation debris was mixed with sediment from elsewhere within and outside the cave. Some of the pedoclasts could have been introduced inadvertently by people (on items of dressing, feet, foodstuffs, etc.). Postdepositional bioturbation by arthropods and plant roots did not completely erase depositional structures. |
| **Upper Layer 6**, correlative with upper **008**: *Two layers of poorly sorted, heterogeneous beige-brown sandy loam with pisoliths, bone and shell fragments.* ***MSB 1*** *separates these from the overlying deposits ( ̴PYS 5)* |
| **FABRIC •Microstructure:** Massive (cm–mm), disrupted by channels and chambers. Also granular (500–50 μm) in the upper layer, immediately below MSB1. Fining-upward trend in the 2 cm below MSB1.  •**Porosity:** 15–25%, dropping to 5–10% near MSB1. Pores: filled root channels; irregular to multiconcave vughs; fissures; intraggregate/intraskeletal pores. •**CFRD:** Open to close porphyric to enaulic at various scales; also chitonic.    **GROUNDMASS** Densely packed, but aggregated at a very fine scale (~30 μm). Beige-brown (PPL); rusty brown, moderately rubified (OIL); with speckled to locally calcite-crystallitic b-fabric (XPL). Root channel fill is strongly crystallitic, reflecting an abundance of calcite. More strongly rubified particles make up 10–20% of the groundmass.    **MINERAL CONTENT •COARSE: Quartz:** 15–20%, dropping to 7–12% near MSB1. Morphologically and optically diverse grains (as in underlying contexts), with many well-rounded grains exhibiting shadowy extinction. Apparently, a bimodal grain population (*cS* and *mS*). MS: 875 μm (lower layer); 2800 μm (base of upper layer). **Other non-carbonate minerals:** Very rare (< 1%): silicified mudstone/chert(?) (*G*); feldspar ruiniform within ferricrete; indeterminate green pleochroic mineral (*fS*); other indeterminate minerals. **Fe-Mn pisoliths/ferricrete:** ~10% (lower layer); 30–35% (upper layer). Compositionally and texturally diverse. Well sorted in the upper layer. Generally, *s–G* (MS: 3840 μm).  **Limestone:** Rare (≤ 2%); mainly angular, often intensely karstified (*cS–G*). **Speleothem:** Rare (≤ 2%); mainly well-rounded, *S–G* (MS: 6080 μm). Also including algal speleothem. •**FINE**  **Quartz silt:** 2–6% (lower layer) to ≤ 2% (upper layer); mainly angular *cs*. **Carbonate silt:** present throughout, in variable concentration. Largely ash-derived?    **BIOGENIC CONTENT •COARSE: Bone:** 3–5% (lower layer) to 10–15% (upper layer: one of the highest contents in the sample set), of which ~10% is burnt. Very diverse in optical properties and state of preservation. From *G* to *S*, with many diminutive, *fS* splinters (MS: 1.5 cm). Many fragments are encased in ash or typic+ash coatings (especially in the upper layer). Both robust and gracile (microvertebrate-derived?) remains. **Shell**: ≤ 2% (lower layer), to 5–10% (upper layer). Mainly angular, diminutive, S-sized fragments. **Charcoal:** ≤ 2% (lower layer), to 2–5% (upper layer). *fS–G*. **Ash:** 2–5%; present as both intraclasts and, possibly, as calcite mixed with the mineral matrix. **Plant tissue/pseudomorphs:** present within ash intraclasts; also as angular humified(?) fragments (*mS*; MS: 720 μm). **Other burning residue:** One porous, opaque particle (1100 μm), with charred organic matrix encasing limestone clasts and bone—resin or fat. **Coproliths:** 2%. Diverse, *cS–G.* Bird or microvertebrate(?) pellets with bone fragments; also rare phosphatic (carnivore–omnivore) coproliths with < 1% organic *s* and dendrites. **Artefacts:** One quartz flake with conchoidal fracture near MSB1: possibly lithic knapping refuse (*G*). •**FINE: Carbonised organic silt**: 1–2% (lower layer) to 2–5% (upper layer). Both in ash intraclasts and dispersed in the groundmass. **Phytoliths:** Rare, within ash intralcasts.    **PEDOFEATURES Typic aggregates:** Less common than in underlying samples. **Typic grain coatings:** Common in aggregated parts; usually anisopachous, as in underlying contexts. **Other:** Rare, disrupted arthropod burrow linings. **Papule, pedorelics, intraclasts:** Common, variable, as in the two underlying units. |
| **INTERPRETATION:** A subtle microstratigraphic transition correlative with the Layer 5/6 boundary in the sampled profile. Algal speleothem particles indicate that the chamber was already unroofed. In both ~basal Layer 5 and ~top Layer 6, much of the sediment content is human occupation debris (food remains, fuel and tool-knapping refuse) reworked by cave-floor processes, perhaps at some distance from foci of human activity (hearths, cooking sites, rake out)? Horizontal particle orientation and fining-up depositional cycles above and below MSB1 may indicate deposition by weakening waterflow (consistent with sheetwash on the cave floor). Ash intraclasts may indicate that hearths (or hearth rake-out deposits) were exposed and partially cementation of for some time before their disruption and reworking. Human occupation debris was mixed with sediment from elsewhere within and outside the cave (as indicated by the pedoclasts). Some of the pedoclasts could have been introduced inadvertently by people, on items of dressing, feet, foodstuffs, etc.). The higher concentration of bone, shell, burnt residues, and knapping flakes in the MSB1 may indicate the formation of a floor between episodes of sheetwash. Postdepositional bioturbation by arthropods and plant roots was extensive, but vestiges of the original depositional structure (e.g., fining-up cycles, imbrication) were preserved. |
| **M13 (PYS 7/8): layer 7/8 boundary**, correlative with the context **010/011** boundary |
| **Basal PYS 7**, correlative with **basal 010:** *Poorly sorted, very heterogeneous brown–beige sandy gravely loam with bone, shell, and ash intraclasts.* *Above textural boundary* ***MSB2*** *(~PYS 7/8 boundary): a zone of comparatively larger grain size, poorer sorting, and lower porosity that separates this from the underlying uni* (~top PYS 8).*t.* |
| **FEBRIC •Microstructure:** Densely packed granular; also weakly developed blocky angular. Poorly sorted throughout, unlike the underlying unit  •**Porosity:** 7–15%; Pores as in the underlying unit; also fissures. •**CFRD:** Double to close porphyric to enaulic at various scales. Also chitonic locally.    **GROUNDMASS** As in the underlying unit. More intensely rubified particles: 15–25%.    **MINERAL CONTENT •COARSE:** As the underlying unit, but overall coarser and more diverse. **Quartz:** 10–12% (MS: 4000 μm). **Non-carbonate rock fragments:** One angular *G* of metagranite/quartzite (with feldspar, and chlorite). **Fe-Mn pisoliths/ferricrete:** More abundant (10–20%), and coarser-grained (*G*) than in the underlying unit. MS: 1.5 cm. •**FINE: Quartz silt:** ≤ 2%; generally *cs*. **Carbonate silt:** present throughout.    **BIOGENIC CONTENT •COARSE:** Overall, as in the underlying unit, but with fewer faunal remains and charcoal. **Bone:** < 2–5%, of which ~10% burnt. **Shell**: 2–3% (up to 10% locally), in diverse states of preservation. *mS* to cm-sized. MS: 5000 μm. **Charcoal:** < 2%. **Plant tissue/pseudomorphs:** Common; humified to partly charred fragments, mainly within ash intraclasts. MS: 3780μm. **Ash:** 2–5%; mainly as intraclasts (MS: 8000 μm). **Coproliths:** 7% - more frequent than the underlying unit. **Artefacts:** Two quartz flakes with conchoidal fracture may be lithic knapping refuse (6000 and 2400 μm).  •**FINE:  Carbonised organic silt**: 2–4%. **Phytoliths:** As in the underlying unit.    **PEDOFEATURES:** As in the underlying unit. In addition, alteration of bone to calcite**.** |
| **INTERPRETATION** Reworked human occupation debris (food remains, fuel and tool knapping refuse) and cave fauna excrement mixed with colluvial sediment matrix. The agent of redeposition is unknown, but may have included water flow (density currents). Alternatively, this could be a rake out deposit. Limited cementation by dripwater, and phosphatisation (from excrement-derived P?) post-deposition. Bioturbation by insects and plant roots. |
| **PYS 8 (upper), correlative with upper 011:** *Poorly (to moderately) sorted brown–beige sandy loam with bone, shell, and ash intraclasts. Separated from the overlying deposit by MSB2.* |
| **FABRIC Microstructure:** Densely packed granular (lower part) to massive (upper part, near MSB2). The *cS* fraction appears moderately sorted. **Porosity:** 5–10%, dropping to ≤ 5% near MSB2. Pores: mainly grain/aggregate packing pores; irregular chambers; various types of intraggregate/intraskeletal pores. **Coarse/Fine-Related Distribution:** Double to close porphyric (to enaulic) at various scales. Also chitonic locally.    **GROUNDMASS** Only moderately aggregated, in contrast to the underlying contexts. Beige-brown to light beige (PPL); speckled to variously intense calcitic-crystallitic b-fabric (XPL); rusty reddish to vitreous olive (OIL), reflecting the relative abundance of calcite (lowest in the upper parts, immediately beneath MSB2). More intensely rubified particles: 15–25%.    **MINERAL CONTENT COASRE: Quartz:** 10–15%, to 7–10% in MSB2. Morphologically diverse grains, from moderately rounded to pitted and ferruginised, indicative of multiple provenance. Probably a bimodal population (*cS* and *mS*). MS: 1375 μm. **Other non-carbonate minerals:** rare (< 2%) feldspar, *fS*. **Non-carbonate rock fragments:** One well-rounded, amber siliceous(?) particle; *cS*. **Fe-Mn pisoliths/ferricrete:** 7–15%. Diverse compositionally and texturally (s–G; MS: 3200 μm). Subhorizontal pisolith clusters define a distinct horizon. **Limestone:** Rare (≤2%); *cS–G*. **Speleothem:** Rare (≤2%); mainly *G–cS*, including algal speleothem.  **FINE:** **Quartz silt:** < 2%; generally *cs*. **Carbonate silt:** present throughout.    **BIOGENIC CONTENT COARSE:** **Bone:** 2–7%, of which ~10% burnt. Various optical properties and preservation state (as in underlying contexts). From *G* to *S*, but mainly *cS–mS*-sized angular splinters. MS: 1.5 cm. Larger bone fragments are clustered together with shell and ash intraclasts.  **Shell**: 2–5% (up to 10% locally). Diverse states of preservation. *mS* to cm-sized; MS ≥ 2.2 cm. **Charcoal:** 2–5%, to ~20% within ash intraclasts. Both ‘free’ particles and encased in ash intraclasts. Generally, *mS–fS;* MS: 3500 μm. **Plant tissue:** Common: humified to partly charred fragments, mainly within ash intraclasts. MS: 3780 μm. **Amorphous charred organics:** Present; well rounded, *S*-sized charred particles of uncertain identification: possibly resin or fat. **Ash:** 2–5%; present as moderately to well-rounded intraclasts (MS: 1 cm), containing phytoliths, charcoal and other plant debris, and druses and plant cell pseudomoprhs (probably derived from leaf burning). Clusters of non-aggregated, dispersed calcite lozenges may also be ash-derived. **Coproliths:** 2%. Diverse: *cS–G*, bird or microvertebrate pellets with bone fragments; also rare phosphatic (carnivore/omnivore) coproliths with < 1% charcoal *s* and dendrites. **Artefacts:** One quartz flake with conchoidal fracture within MSB2 may be lithic knapping refuse (6000 μm).  **FINE: Carbonised organic silt**: 2–4%, dropping to ≤ 2% in MSB2. **Phytoliths:** Present (to abundant) within ash intraclasts and in other calcite (ash)-rich parts.    **PEDOFEATURES Typic aggregates:** Less common than in underlying units. **Typic grain coatings:** In aggregated, granular parts: (1) reddish-brown silt (typic groundmass); (2) calcite lozenge coatings. Where coating successions are present, coatings (2) succeed coatings (1). **Other:** Rare, disrupted arthropod (termite?) burrow linings. **Papule, pedorelics, intraclasts:** Common, variable: (1) well-rounded ash intraclasts (up to 1 cm), as described; (2) compound aggregates, with typic aggregates + pisoliths + quartz ± other mineral *S* +shell, cemented with clear microspar (grain- to cement-supported) – possibly derived from cemented cave ground; (3) rounded to mammillate, yellow to strongly rubified clay + quartz s ± ≤2% charcoal silt – possibly a pedoclast (?).  **Fe-Mn oxide:** Common hypocoatings around and inside aggregates (to incipient nodules). **Organic:** Soot in intraskeletal pores and around calcined plant tissue pseudomoprhs (in ash). **Calcareous:** Clear microspar cement around typic groundmass aggregates and within bone pores. **Isotropic (Phopshatic?):** Patchy phosphatisation of ash calcite (rare). |
| **INTERPRETATION** Redeposition of human occupation debris (food remains, fuel) by cave floor processes and/or by human activity. Some of the ash intraclasts and diffuse ash content may have eroded from the adjacent ash ‘lamp’ and surrounding ash deposits and/or they may represent (further) reworking of raked out/swept ashes. Burning residue indicates the use of (mainly) non-woody fuel (leaves?).   As with overlying deposits, mixing with sediment from elsewhere within and without the cave (but some of the pedoclasts – soil fragments could have been human introductions).   Preservation of coherent ash intraclasts may indicate exposure and cementation of hearths or raked out/swept ashes for some time before reworking.   Limited cementation by dripwater and phosphatisation (excrement-derived P?)   Bioturbation by insects and plant roots. |
| **M10_PYS 8/9:** Layer 8/9 boundary, correlative with the boundary between context **011** and the underlying talus. |
| **PYS 8 (basal), correlative with basal 011:** *Poorly sorted sandy pisolithic loam with bone, shell, charcoal and diverse intraclasts. A weak fining-up trend is present locally.* |
| **Microstructure:** As in PYS 9 (upper); also moderately sorted, fining up-domains.  **Porosity:** Very variable: 7–25%. Pores as in PYS 9(upper), but without low-angle fissures. **Coarse/Fine-related distribution:** Single to open fine enaulic; locally chitonic.    **GROUNDMASS:** As in PYS 9 (upper), but without distinct ash-rich domains. More intensely rubified particles: 10–20%.    **COARSE MINERAL CONTENT:** Overall as in PYS 9(upper). **Quartz**: 4–6%. MS: 4000μm (but this could be tool knapping refuse). **Fe-Mn pisoliths/ferricrete:**  3–5% (G-sized); 10–12% (S-sized).    **FINE MINERAL CONTENT:** **Quartz silt:** <2 %, but somewhat more abundant than in PYS 9(upper). **Calcite silt:** as in PYS 9 (upper).    **COARSE BIOGENIC CONTENT:** Overall as in PYS 9 (upper)**. Bone:** 5–7%, of which ~10–20% burnt. Often encased in ash coatings. **Shell:** 1–2%, somewhat less abundant than in PYS 9 (upper). **Charcoal:** 5–7%. **Ash intraclasts:** less abundant than in PYS 9 (upper).    **FINE BIOGENIC CONTENT:** As in PYS 9 (upper). **Carbonised oganic silt:** 2–3%. **Phytoliths** and **amorphous organics:** rare.    **PEDOFEATURES:** As in PYS 9 (upper). |
| **INTERPRETATION** Floor colluvium with mixed occupation debris, above activity surface. Sharp increase in coarse biogenic (putative human) inputs from this level up probably indicates intensification of human activity at/near the trench site. Bioturbation has obscured primary stratification, but fining-up domains may indicate deposition by rain-triggered wash. |
| **PYS 9 (top), potentially correlative with the top of the talus:** *Poorly sorted, fining up sandy pisolithic loam with bone, shell, charcoal and diverse intraclasts, topped by an inclined, bioturbated and disrupted layer of ash (separated in incipient intraclasts), charcoal and bone fragments (burnt and unburnt). This horizon* ***(MSB 3)*** *may correlate with the PYS 8/9 boundary.* |
| **Microstructure:** Channel (cm); granular to crumb (> 1000–40μm). Also, poorly to moderately developed blocky angular within intraclasts. Rare, isolated patches of (almost) monic microstructure. A fining-up trend is evident in the upper 3 cm of the unit. Pisoliths and other G-sized particles are moderately sorted in the 1500–2500-μm range.  **Porosity:** 20–30%, dropping to 10–15% in MSB3 (immediately below the ash deposit). Pores: large channels (≥ 1.9 cm), intergranular/interaggregate packing pores (predominant); low-angle accommodating fissures parallel to MSB3 (≤ 350 μm); multiconcave vughs;  intraggregate fissures/channels; skeletal pores. **Coarse/Fine-related distribution:** Single to open fine enaulic at various scales; aslo porphyric around MSB3.    **GROUNDMASS:** Thoroughly aggregated (ovoid to mammillate aggregates) at various scales. PPL: orange, to grey in MSB3. XPL: speckled to variably calcitic-crystallitic (reflecting the relative abundance of ash calcite); calcitic-crystallitic in MSB3. OIL: rusty brown, moderately rubified, to vitreous grey MSB3. More strongly rubified particles: 10–20%, rising to ~30% in MSB3.    **COARSE MINERAL GRAINS:** **Quartz:** 5-10%. Moderately sorted, perhaps bimodal, with cS and mS subpopulations (~480 and 240 μm). Both angular and well rounded, ferruginised and non-ferruginised grains. **Other non-carbonate minerals**: < 2%. Perhaps the most variable in the sample set, including **Andalusite/sillimanite**(?) (angular, cS–mS), **Feldspar** (angular, S), a green, pleochroic, low-birefrigence mineral (**corundum**?) (very rare), and **other indeterminate** minerals (1^st^-order birefringence colors).  **Non-carbonate rock fragments:** One yellow siltstone particle – moderately rounded, G. **Fe-Mn pisoliths/ferricrete:** Mainly G (3–5 %; apparently moderately sorted) to mS (10–15%, to 7–10% around MSB3).  **Limestone:** ≤ 2%. Moderately rounded to angular, cS. **Speleothem**: ≤ 2%. Mainly angular, irregular, cS; including laminated micritic (algal?) speleothems.    **FINE MINERAL CONTENT: Quartz silt:** < 1%. **Calcite silt:** present throughout and locally abundant. Perhaps much of it is ash-derived.    **COARSE BIOGENIC CONTENT:** **Bone:** 5–7%, dropping to ~2% in MSB3. Diverse optically (from clear/pale yellow to deep orange – burnt), taxonomically (both gracile (from cave microvertebrates?) and robust fragments), and in state of preservation (with beige, altered bone). Both very angular splinters and well-rounded fragments. Generally, cS–mS splinters to cm-scale fragments. Burnt bone: 10– 20% of the total bone, rising to ~50–80% of the total bone within MSB3. **Shell:** 2–3%; generally cS–mS, in diverse states of preservation (from unaltered to fissured/degraded and coated fragments). **Charcoal:** ≤ 2%, rising to 5–30% within MSB3 (where it is especially abundant within ash intraclasts). From G (wood charcoal) to fS (predominantly). Most particles appear to be derived from non-woody tissue(?). **Ash:** Abundant along MSB3, as incipient intraclasts (containing plant tissue pseudomorphs and phytoliths); also as isolated calcite ash aggregates. **Non-charred vegetable tissue/organs:** Present (to common) within ash intraclasts in MSB3. **Coproliths:** Rare (≤ 2%). : cS to G. Diverse: bird or other small animal feces and other porous phosphatic coproliths (very rare).    **FINE BIOGENIC CONTENT: Carbonised oganic silt:** 2–3%; up to 10% in MSB3. **Phytoliths:** Present (to abundant) within ash intraclasts, especially in MSB3. **Amorphous organics:** present within ash intraclasts (especially in MSB3).    **PEDOFEATURES:** **Typic aggregates:** Abundant, as described. **Typic grain coatings:** Common, of two main types: (1) Reddish silty loam (±Fe oxide impregnates); (2) Coalescent calcite lozenges (ash-derived). **Papule, pedorelics, intraclasts:** Abundant; very variable: (1) Yellow/red striated clay with ~7% quartz S: up to G-sized. Probable pedoclasts; (2)Orange clay + pisoliths+ quartz + charcoal silt (~1–2%) ± shell, with blocky angular microstructure: Rare. MS: 3200μm. Pedoclast/intraclast;  (3)  Ash intraclasts, with calcite lozenges (ash) + quartz sand+ rip-up clasts ± bone, shell, phosphatic(?) neomorphs: these intraclasts define MSB3. MS: 1.2 cm; (4) Microlaminated micritic calcite + well-sorted quartz silt. Sediment crust. Rare; (5) Strongly rubified angular intraclasts with poorly sorted, angular quartz (cS–fS + bone + Fe_2_O_3_/organic impregnation. In MSB3. Reworked subhearth deposit? (6) Laminated phosphate/nitrate/Mn/Fe oxide intraclast. Rare. Probably an organomineral/phosphatic crust? (7) Well rounded compound aggregates (typic + pisoliths + quartz S) cemented with clear microspar cement. Probably fragments of a cemented cave-floor crust? (8) Dense, grain-supported quartz S in red clay matrix. cS-sized. Rare. Pedoclast or reworked cave-floor crusts.  **Fe/Mn oxide hypocotings:** In fissures, around mineral (e.g. quartz) and bone particles, and within ash calcite mosaics. Probably chelated, Fe oxide + organic deposits.  **Calcareous:** Clear equant microspar cement. Also, interlaced acicular crystals (≤ 12.5 μm) – probably aragonite. **Isotropic (phosphatic?):** common, variable. Phosphatisation of shell, calcite crystals and ‘silt crust’ surface. Also, phosphate nodules within ash intraclasts. |
| **INTERPRETATION** Reworked hearth waste on a gently inclined palaeofloor formed on in-wash colluvium. Fuel comprised predominatly non-woody tissue.  The underlying colluvium contains abundant human habitation debris mixed with mineral matrix.  High quartz content and diverse silicate minerals may signal increased sediment influx from outside the cave. The presence of delicate (inferred) sediment crusts indicates (rapid?) reworking of cave floor(s) into this colluvium. Pervasive bioturbation and phosphatisation (with P probably derived from cave fauna excrement and human inputs). |
