# Supplementary figures and images for: Cave morphology and human-mediated sediment deposition: Late Pleistocene to Holocene evolution of the cave floor at Panga ya Saidi, coastal Kenya
Source: PLoS One. 2026 May 20;21(5):e0347491. doi: 10.1371/journal.pone.0347491 (PMC13189332; doi:10.1371/journal.pone.0347491)

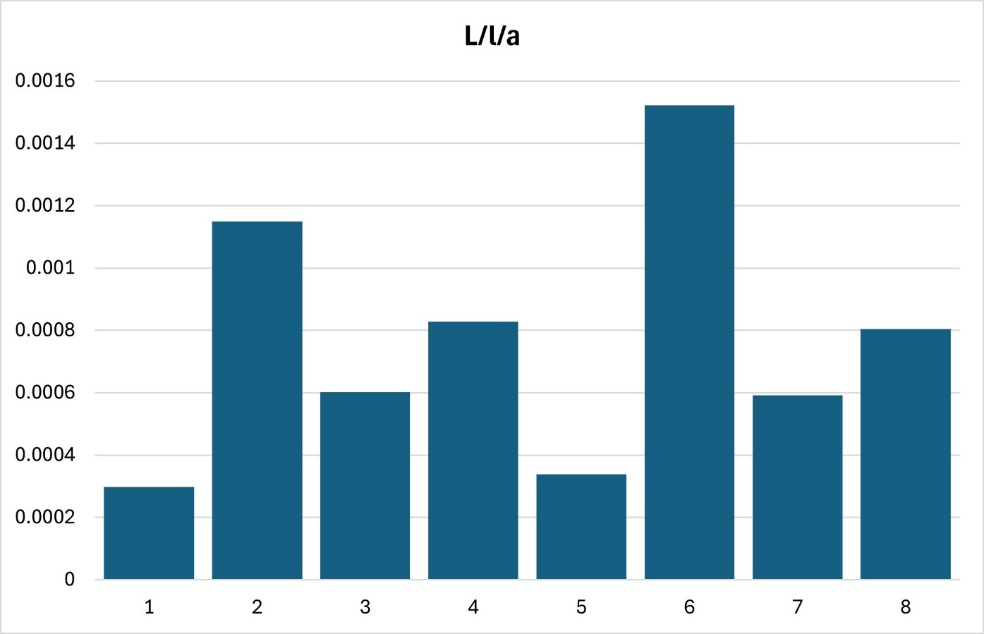


**S1 Figure. Lithics per liter per year for Layers 1-8 at PYS.**

Supplement: S1 Fig — (DOCX) [file pone.0347491.s005.docx]
